# Life-Long Genetic and Functional Access to Neural Circuits Using Self-Inactivating Rabies Virus

**DOI:** 10.1016/j.cell.2017.06.014

**Published:** 2017-07-13

**Authors:** Ernesto Ciabatti, Ana González-Rueda, Letizia Mariotti, Fabio Morgese, Marco Tripodi

**Affiliations:** 1 Division of Neurobiology, MRC Laboratory of Molecular Biology, Cambridge, UK

**Keywords:** neural circuits, rabies, circuit manipulations, network activity, optogenetic, in vivo imaging, transsynaptic tracing

## Abstract

Neural networks are emerging as the fundamental computational unit of the brain and it is becoming progressively clearer that network dysfunction is at the core of a number of psychiatric and neurodegenerative disorders. Yet, our ability to target specific networks for functional or genetic manipulations remains limited. Monosynaptically restricted rabies virus facilitates the anatomical investigation of neural circuits. However, the inherent cytotoxicity of the rabies largely prevents its implementation in long-term functional studies and the genetic manipulation of neural networks. To overcome this limitation, we developed a self-inactivating ΔG-rabies virus (SiR) that transcriptionally disappears from the infected neurons while leaving permanent genetic access to the traced network. SiR provides a virtually unlimited temporal window for the study of network dynamics and for the genetic and functional manipulation of neural circuits in vivo without adverse effects on neuronal physiology and circuit function.

## Highlights

• Self-inactivating rabies virus (SiR) provides life-long access to neural networks
• SiR has no adverse effects on neuronal physiology and network computations
• SiR allows open-ended Ca^2+^ imaging of network activity and functional intervention
• SiR allows generation of circuit-specific knockouts

## Graphical Abstract

**Figure:**
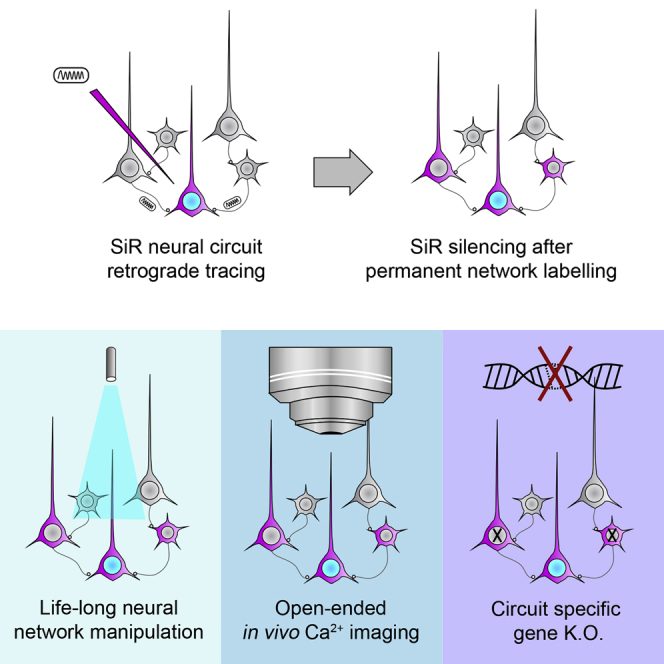

## Teaser

A self-inactivating rabies virus provides a virtually unlimited temporal window for the study and manipulation of neural circuits in vivo without adverse effects on neuronal physiology and circuit function.

## Introduction

Network dynamics are thought to be the substrate of brain information processing and of mental representations (Buzsáki and Freeman, 2015, McIntosh, 2000). From a clinical perspective, network-wide dysfunction is also beginning to be recognized as the culprit behind a number of psychiatric (Rubinov and Bullmore, 2013) and neurodegenerative disorders (Roselli and Caroni, 2012). Consequently, major efforts have been made in the development of methods to unravel the organization of neural networks both in animal models and humans (Callaway, 2008, Cardona et al., 2010, Livet et al., 2007, Osten and Margrie, 2013, van den Heuvel et al., 2016). However, defining circuit architecture is only the first step toward understanding the physical implementation of information processing in the brain. Information about circuit topology, namely the relationships among neurons within a connected network, should also be used to gain functional and genetic access to topologically defined network elements, as this would allow probing their functional role in neural computation and behavior.

The recent development of G-deleted rabies virus (ΔG-rabies) (Mebatsion et al., 1996, Wickersham et al., 2007b) provided the first non-electron microscopy (EM)-based method aimed at revealing network synaptic topology upstream of defined nodes (Miyamichi et al., 2011, Stepien et al., 2010, Tripodi et al., 2011, Wickersham et al., 2007b). Despite the transformative role of ΔG-rabies-based approaches in the anatomical investigation of neuronal circuits, their use to manipulate the functional properties of neural networks in vivo remains limited. The main reason of this limitation relates to the cytotoxic effects of the rabies virus. The ΔG-rabies is a replication-competent virus that eventually leads to neuronal death (Wickersham et al., 2007a). The temporal window for optical imaging and functional interventions enabled by ΔG-rabies in functionally unaffected circuits is thus limited to 5–17 days from infection (Namburi et al., 2015, Tian et al., 2016, Wertz et al., 2015).

We reasoned that, in order to circumvent this limitation, it would be sufficient to transcriptionally silence the virus shortly after the primary infection. Transcriptional silencing of DNA-based viruses, such as adeno-associated viruses (AAVs), is generally achieved by using conditional recombinant cassettes that invert the viral genes of interest upon recombination (Atasoy et al., 2008). However, strategies to conditionally manipulate the expression of RNA-based viruses, such as rabies, are currently lacking. Given the unique coupled nature of transcription and replication of the rabies virus and their dependence on virally encoded proteins (Finke and Conzelmann, 2005), we hypothesized that the conditional modulation of viral protein stability, as opposed to viral genome recombination, could act as an ON-OFF switch with which to direct the viral transcription-replication cycle. To this aim, we engineered the rabies genome so that selected viral proteins could be reversibly targeted to the proteasome using a conditionally cleavable proteasome-targeting domain. This gave origin to a self-inactivating ΔG-rabies (SiR) that switches OFF following the primary infection, thereby preventing cytotoxicity while providing permanent genetic access to the mapped neural elements via a CRE/FLP-mediated recombination event triggered soon after the infection. We show that SiR-infected neurons retain unaltered physiological properties, functional connectivity, and normal synaptic function several months following the primary infection and, likely, for the entire life of the animal. Furthermore, we used SiR to perform in vivo 2-photon calcium imaging of V1 neurons projecting to V2, showing that computational properties of V1 neurons, such as their orientation tuning, remain intact after SiR infection. The development of the SiR virus gives, for the first time, permanent functional and genetic access to neural networks with no adverse effects on neural physiology, circuit function, and circuit-dependent computations.

## Results

### Conditional Control on the Rabies Life Cycle

**Figure 1:**
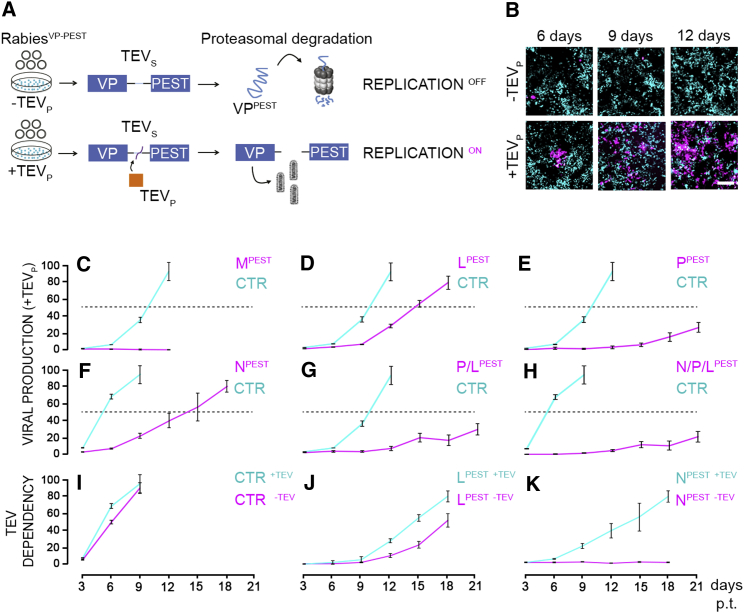
Screening Viral Amplification Efficiency after Systematic Proteasome Targeting of Viral Proteins (A) Reversible viral protein (VP) destabilization via proteasome targeting PEST domain through TEV protease (TEVp) cleavage of TEV site (TEVs). (B) TEV_P_-dependent viral amplification (magenta) in HEK-GG (−TEVp) and HEK-TGG (+TEVp). Scale bar, 200 μm. (C–H) Quantification of amplification efficiency for all recombinant rabies constructs (magenta) and control rabies (cyan) as a percentage of infected cells (mCherry positive) (mean ± SD; dashed line shows 50% threshold level used in the screening). M^PEST^ (C), L^PEST^ (D), P^PEST^ (E), N^PEST^ (F), P/L^PEST^ (G), and N/P/L^PEST^ (H) are shown. (I–K) Quantification of amplification efficiency in HEK-TGG (cyan, +TEVp) and HEK-GG (magenta, −TEVp). x axis, days post-transfection (p.t.); y axis, amplification efficiency. CTR (I), L^PEST^ (J), and N^PEST^ (K) are shown. See also Figures S1, S2, S3, and Table S1.

Given that both transcription and replication of the rabies virus depend on virally encoded proteins, we hypothesized that conditional modulation of viral protein stability may act as a switch on the viral transcription-replication cycle. To achieve such a modulation, we fused a PEST proteasome-targeting domain to each and every protein of the ΔG-rabies virus (individually or in combinations) in order to direct their proteasomal degradation (Figure 1A; full list of constructs in Table S1). To implement a level of conditional control on viral protein stability, the Tobacco Etch Virus cleavage site (TEVs) was interposed between the particular viral protein and the proteasome-targeting domain. The tobacco etch virus protease (TEVp) selectively cleaves the TEVs linker, separating the viral proteins from the proteasome-targeting domain, sparing them from degradation (Figures 1A and 1B). The binary system composed by TEVp and TEVs should provide a form of exogenous control on the extent and temporal window of viral protein degradation during viral production and, potentially, in vivo. Namely, the virus should be able to transcribe and replicate only when TEVp is present, giving origin to a system in which viral transcription and replication are constitutively OFF unless TEVp is provided.

**Figure S1:**
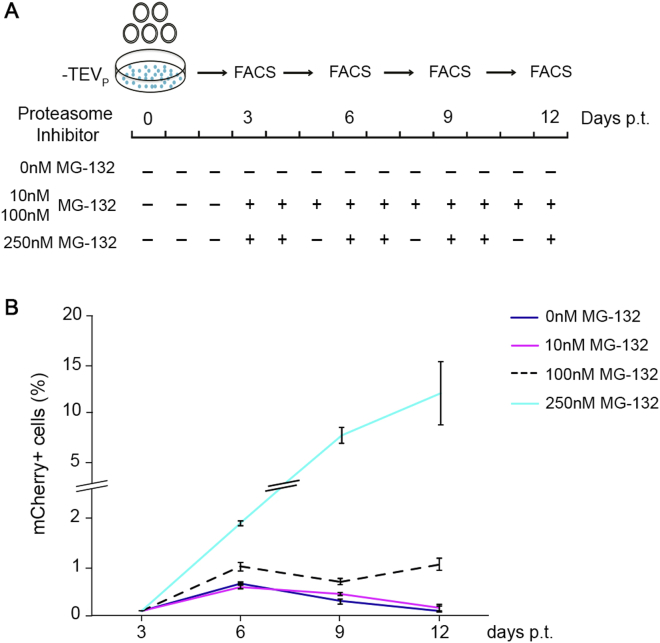
Proteasome Inhibition Supports Attenuated Rabies Production, Related to Figure 1 (A) Scheme of tested conditions for the production of N-tagged ΔG-Rabies^mCherry^ in HEK-GG (-TEV_P_) cells in presence or absence of proteasome inhibitor (MG-132). (B) Percentage of infected cells at different concentrations of MG-132 administered after 3-12 days post-transfection (mean ± SEM, n = 3).

**Figure S2:**
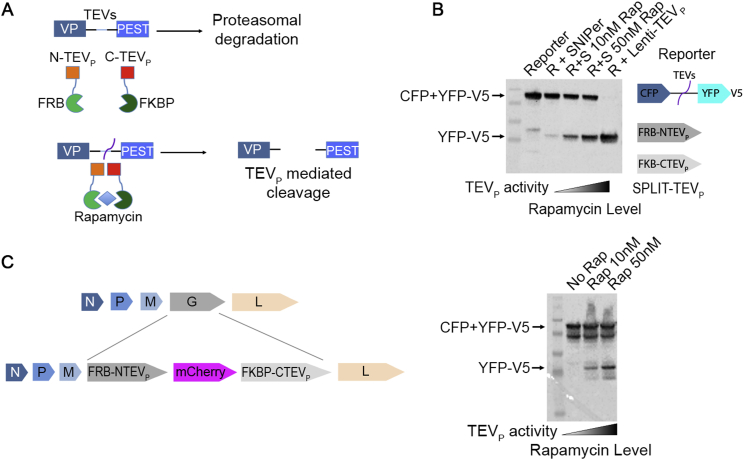
Rapamaycin-Induced SPLIT-TEVp Reconstitution and Cleavage of PEST Domain in HEK293T Cells, Related to Figure 1 (A) Strategy for the pharmacological stabilization of tagged viral protein by rapamycin-induced dimerization of the SPLIT-TEVp. (B) SPLIT-TEVp rapamycin response in HEK293T cells transfected with a TEVp activity reporter increases at incremental concentration of rapamycin (0-10-50 nM). (C) The SPLIT-TEVp cassette was cloned into the glycoprotein locus in the Rabies genome. Rapamycin dependent TEVp activity in HEK293T 48 hr post transfection with TEVp activity reporter and infection with the SPLIT-TEVp expressing ΔG-Rabies.

**Figure S3:**
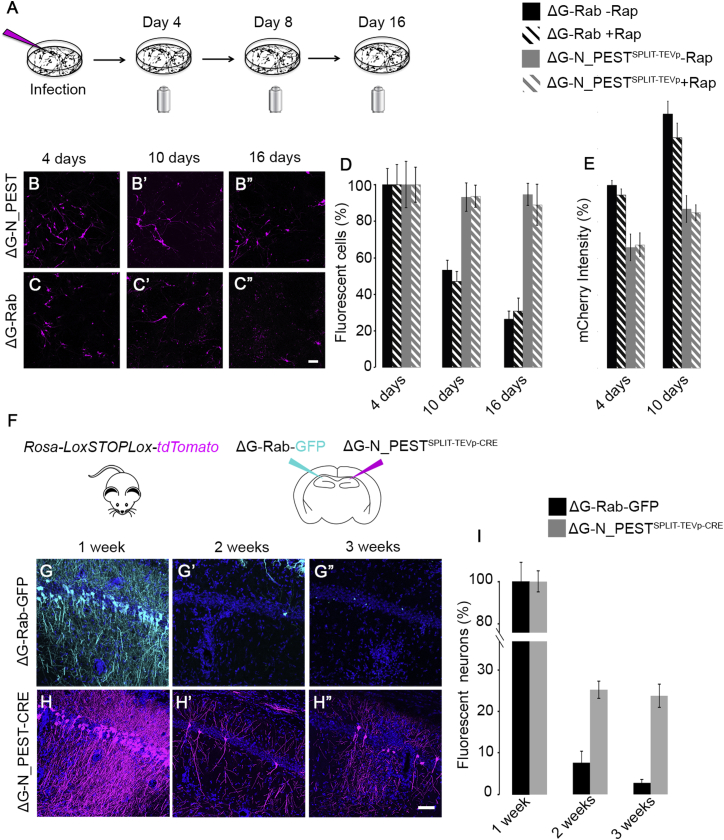
Testing Cytotoxicity of ΔG-N^PEST^Rabies^SPLIT-TEVp-mCherry^ In Vitro and In Vivo, Related to Figure 1 (A) hESCs derived neurons were infected with ΔG-N^PEST^Rabies^SPLIT-TEVp-mCherry^ and imaged longitudinally over 16 days. (B)-B”) ΔG-N^PEST^Rabies^SPLIT-TEVp-mCherry^ and B19 ΔG-Rabies control (C)-C”) infected hESCs derived neurons imaged at 4, 10 and 16 days post-infection (p.i.). (D) Percentage of infected cells after administration of control ΔG-Rabies or ΔG-N^PEST^Rabies^SPLIT-TEVp-mCherry^ in presence or absence of rapamycin after 4–10 and 16 days normalized to day 4 time-point (mean ± SEM). (E) mCherry signal intensity of ΔG-N^PEST^Rabies^SPLIT-TEVp-mCherry^ and ΔG-Rabies infected neurons normalized to day 4 time-point (mean ± SEM., scale as in (D). Scale bar: 50 μm. (F) Hippocampi of Rosa-*LoxP-STOP-LoxP-YFP* mice were injected bilaterally with ΔG-Rabies^GFP^ (cyan) and ΔG-N^PEST^Rabies^SPLIT-TEVp-CRE^ (magenta). Confocal images of ΔG-Rabies^GFP^ (G-G’) or ΔG-N^PEST^Rabies ^SPLIT-TEVp-CRE^ (H-H’) infected hippocampi at 1, 2 or 3 weeks p.i. Scale bar: 50 μm (I) Percentage of infected neurons at 1, 2 or 3 weeks p.i. of ΔG-Rabies^GFP^ (black) or ΔG-N^PEST^Rabies^SPLIT-TEVp-CRE^ (gray) in hippocampus normalized to 1 week time-point (mean ± SEM, n = 3 animals per time point).

We screened the suitability to viral production and TEVp dependence of six viral constructs, in which each viral protein was targeted to the proteasome alone or in combination (Figures 1C–1K; Table S1). To this aim, we generated a stable cell line expressing TEVp and the spike glycoprotein (Figures 1A and 1B). Surprisingly, the destabilization of the viral proteins M, P, N/P, and N/P/L led to a virus unable to efficiently spread even in presence of the protease. In contrast, the virus with proteasome-targeted L protein did amplify both in presence (Figure 1D) and absence (Figure 1J) of the TEV protease, indicating that the partial destabilization of the L protein by the degradation domain is insufficient to impair the viral replication machinery. The virus in which the N protein alone was destabilized showed the desired conditional control: failure to amplify in absence of TEVp and successful amplification in TEVp-expressing cells (at 18 days post transfection +TEVp 80% ± 4%, −TEVp 2% ± 0.2% of infected cells, p = 3 × 10^−3^, two-tailed two-sample Student’s t test, Figure 1K). This indicates that N is the sole viral protein whose conditional destabilization, in our design, is sufficient to reversibly suppress the viral transcription-replication cycle. We confirmed that the conditional inactivation of the N-tagged ΔG-rabies is a proteasome-dependent process by amplifying the virus in absence of TEVp but in presence of the proteasome inhibitor MG-132 (Figure S1A). Indeed, the amplification rate of the N-tagged ΔG-rabies virus significantly increases, in a dose-dependent manner, following the administration of the proteasome inhibitor MG-132 (at 12 days post transfection 0 nM MG-132, 0.1% ± 0.1%; 250 nM MG-132, 13% ± 5% of infected cells, p = 0.04, two-tailed two-sample Student’s t test, Figure S1B). After a further round of improvement on the viral cassette design (Figures S2 and S3; STAR Methods), we were able to produce a SiR with the desired TEVp-dependent ON/OFF kinetics constituted by an NPEST-rabies-mCherry cassette containing a TEVp-cleavable linker between N and the PEST sequence.

### Permanent Genetic Access to Neural Circuits with SiR

**Figure 2:**
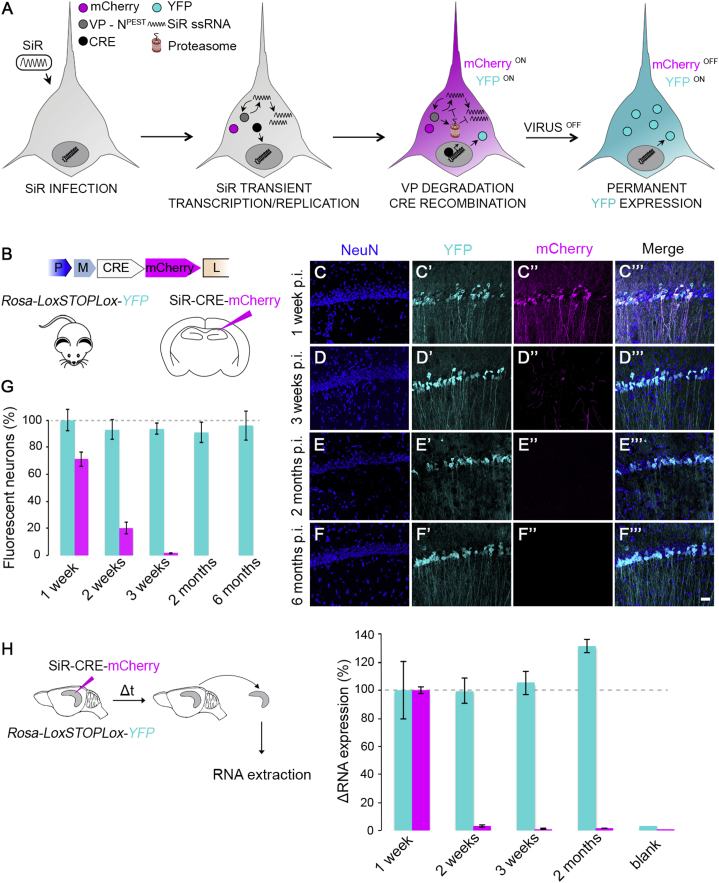
Absence of Cytotoxicity In Vivo (A) SiR life cycle scheme. (B) SiR expression cassette and experimental procedure. (C–F′″) Confocal images of hippocampal sections of *Rosa-LoxP-STOP-LoxP-YFP* mice infected with SiR^CRE-mCherry^ and imaged at 1 week (C–C′″), 3 weeks (D–D′″), 2 months (E–E′″), and 6 months (F–F′″) p.i. Scale bar, 25 μm. (G) Number of YFP and mCherry positive neurons at 1–3 weeks, 2 months, and 6 months p.i. normalized to 1 week time point (mean ± SEM, n = 3 animals per time point). (H) Levels of viral RNA (magenta) and endogenous *YFP* expression (cyan) normalized to 1 week RNA level (mean ± SEM, n = 3 animals per time point). See also Figures S4 and S5.

**Figure S4:**
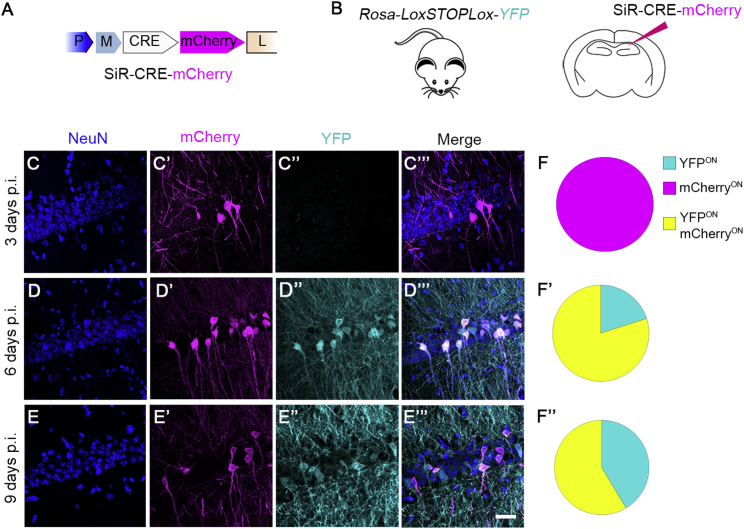
Short-Term SiR^CRE-mCherry^ Kinetics In Vivo, Related to Figure 2 (A) SiR^CRE-mCherry^ cassette design. (B) SiR^CRE-mCherry^ injection in CA1 of *Rosa*-*LoxP-STOP-LoxP-YFP* mice. (C-E”’) Confocal images of CA1 pyramidal neurons infected with SiR^CRE-mCherry^ at 3, 6 and 9 days p.i. Scale bar: 25 μm. (F-F’’) Percentage of YFP^ON^, mCherry^ON^ and YFP^ON^mCherry^ON^ neurons at 3, 6 and 9 days p.i.

**Figure S5:**
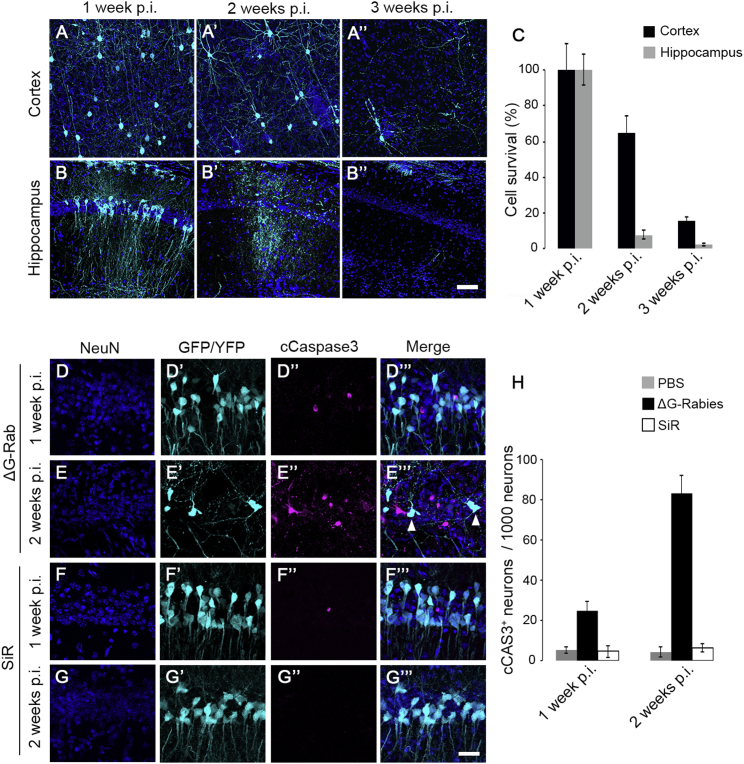
ΔG-Rabies Induced Mortality in Cortex and Hippocampus, Related to Figure 2 (A-A’’) Confocal images of cortical neurons and (B-B’’) CA1 pyramidal neurons infected with ΔG-Rabies^GFP^ at 1, 2 and 3 weeks p.i. Scale bar: 50 μm. (C) Percentage of ΔG-Rabies infected neurons at 1, 2 or 3 weeks p.i. in cortex (black) or hippocampus (gray) normalized to 1 week time-point (mean ± SEM,) (hippocampus, 92% ± 3% cell death at 2 weeks, n = 3 animals per time-point, one-way ANOVA, F = 101, p = 2.4x10^−5^; cortex 85 % ± 2% cell death at 3 weeks, n = 3 animals per time-point, one-way ANOVA, F = 17, p = 3.2x10^−3^). Confocal images of ΔG-Rabies^GFP^ (D-E’’’) or SiR^CRE^ (F-G’’’) infected hippocampi of *Rosa-LoxP-STOP-LoxP-YFP* mice at 1 and 2 weeks p.i. stained for cleaved caspase-3 (cCaspase3) (arrowheads point to ΔG-Rab-cCaspase3 double positive neurons). Scale bar: 25 μm (H) Percentage of positive cCaspase3 neurons every 1000 neurons at 1 and 2 weeks p.i. in PBS, ΔG-Rabies or SiR infected hippocampi (mean ± SEM, n = 3 animals per time-point).

In order to experimentally assess the in vivo cytotoxicity of the newly generated SiR, we added a CRE recombinase and a destabilized mCherry (SiR^CRE-mCherry^, Figure 2B) to the SiR genome. We then tested SiR transcription-replication kinetics and cytotoxicity in vivo by injecting SiR^CRE-mCherry^ in the CA1 pyramidal layer of *Rosa-LoxP-STOP-LoxP-YFP* mice (Figure 2B). The transient expression of the CRE recombinase driven by the SiR should be adequate to ensure a permanent recombination of the Rosa locus and consequent YFP expression even after a complete transcriptional shut down of the virus (Figure 2A). At the same time, the destabilized mCherry marks the presence of active virus with high temporal resolution. Indeed, while at 3 days post infection (p.i.) only the virally encoded mCherry can be detected (Figures S4C–S4C″′ and S4F), by 6 days p.i. the virally encoded CRE has induced recombination of the conditional mouse reporter cassette, triggering the expression of YFP in all infected neurons (Figures S4D–S4D″′ and S4F′). In order to exclude any SiR-induced cytotoxicity, we monitored the survival of SiR^CRE-mCherry^-infected CA1 pyramidal neurons over a 6-month period. By 3 weeks p.i., the SiR had completely shut down (98% ± 2% YFP^ON^ mCherry^OFF^, Figures 2D–2D″′ and 2G) and, more importantly, no significant neuronal loss was observed during the 6-month period following SiR infection (one-way ANOVA, F = 0.12, p = 0.97, Figures 2F–2F″′and 2G). This is in striking contrast with what is observed after canonical ΔG-rabies infection, in which the majority of infected neurons in the hippocampus die within 2 weeks from the primary infection (Figures S5A–S5C). We further confirmed the absence of SiR-induced cytotoxic effects in vivo by assessing the level of caspase-3 activation (cCaspase3) at 1 and 2 weeks following SiR infection and comparing it to that elicited in mock-injected controls. The results indicate no significant differences between SiR and mock-injected conditions in the levels of caspase-3 activation (one-way ANOVA, F = 0.11, p = 0.7, Figures S5F–S5G″′ and S5H). Monitoring the in vivo viral RNA genomic titer during the course of the infection also shows that SiR completely disappears (at a genomic level) from the infected neurons by 2 weeks p.i. (Figure 2H). Overall, these results show that the SiR^CRE-mCherry^ transcription-replication kinetics provides enough time to generate an early CRE recombination event (Figures S4D–S4D″′) before the virus disappears (Figures 2D–2D″′). It thereby ensures permanent genetic (CRE-mediated) access to the infected neurons without affecting their survival (Figures 2G and 2H).

### SiR Pharmacological Reactivation In Vivo

**Figure 3:**
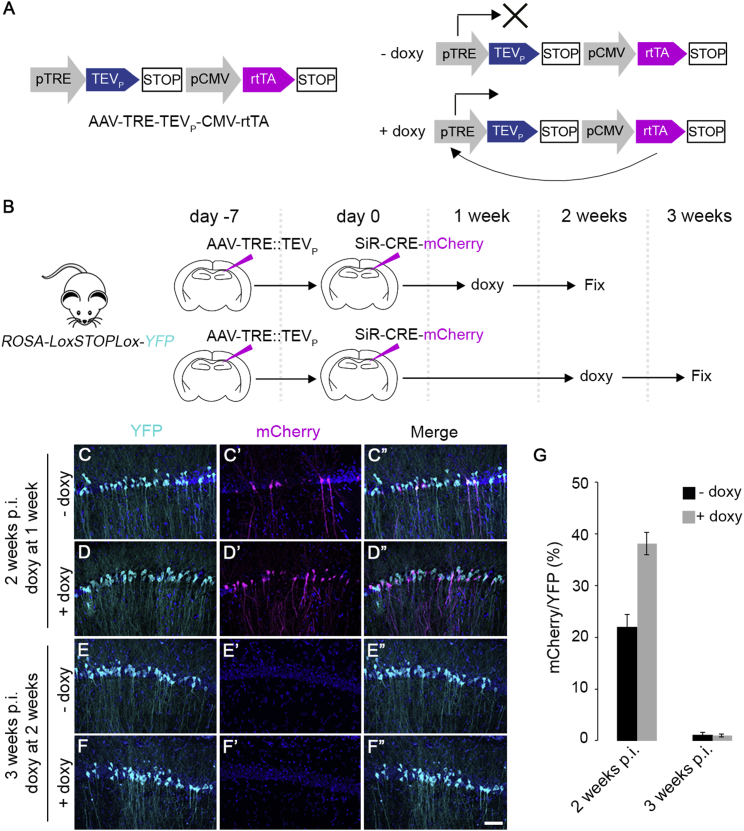
Pharmacological Reactivation of SiR (A) Design of the doxycycline-inducible AAV. The rtTA transactivator is constitutively expressed by the virus and in the presence of doxycycline, it drives the TEV protease expression. (B) Diagram of AAV^TRE::TEVp^ injection in the hippocampus of *Rosa-LoxP-STOP-LoxP-YFP* mice followed by SiR^CRE-mCherry^ injection in the same region 1 week after. Doxycycline was administered at 1 or 2 weeks post SiR infection. (C–F″) Hippocampal pyramidal neurons infected with SiR and AAV^TRE::TEVp^, reactivated with doxycycline at 1 week (D–D′″) or 2 weeks (F–F′″) post SiR infection and untreated controls imaged at 2 weeks (C–C′″) and 3 weeks (E–E′″) p.i. Scale bar, 50 μm. (G) Quantification of mCherry^ON^ neurons over the total YFP^ON^-infected neurons (mean ± SEM, n = 3 animals per time point).

Experiments in vitro indicate that modulation of viral stability by conditional proteasome degradation is sufficient to modulate the viral transcription-replication cycle. In order to assess whether conditional control over viral protein degradation can be achieved in vivo, we designed an AAV virus to express TEVp under a doxycycline-inducible promoter (AAV^TRE::TEVp^, Figure 3A) (Shockett and Schatz, 1996). This should provide pharmacological control on the viral transcription-replication cycle by doxycycline-mediated release of viral N protein from targeted proteasomal degradation.

To address if and at what stage post infection SiR can be reactivated by doxycycline administration, we injected CA1 neurons in the hippocampus with AAV^TRE::TEVp^ followed by the SiR^CRE-mCherry^ injection 1 week after. Doxycycline (100 mg/kg) was then administered by oral gavage for 2 days at two different time points, after 1 or 2 weeks post SiR infection (Figure 3B). In agreement with the viral RNA profile analysis, administration of doxycycline at 1 week post SiR infection, when the virus is still transcriptionally active, significantly increases the percentage of YFP^ON^ mCherry^ON^ neurons (YFP^ON^-mCherry^ON^ −doxy 22% ± 3%, +doxy 38% ± 2%, p = 0.02, Figures 3C–3D″ and 3G). Moreover, we observed no effect by administering doxycycline 2 weeks post SiR infection (YFP^ON^-mCherry^ON^ −doxy 1% ± 1%, +doxy 1% ± 1%, p = 0.2, Figures 3E–3G). These results indicate that the transcriptional activity of the virus, as reflected by mCherry expression, can be sustained in vivo if doxycycline is administered in an early time window before the complete disappearance of the virus.

### SiR Transsynaptic Spreading Capabilities

**Figure 4:**
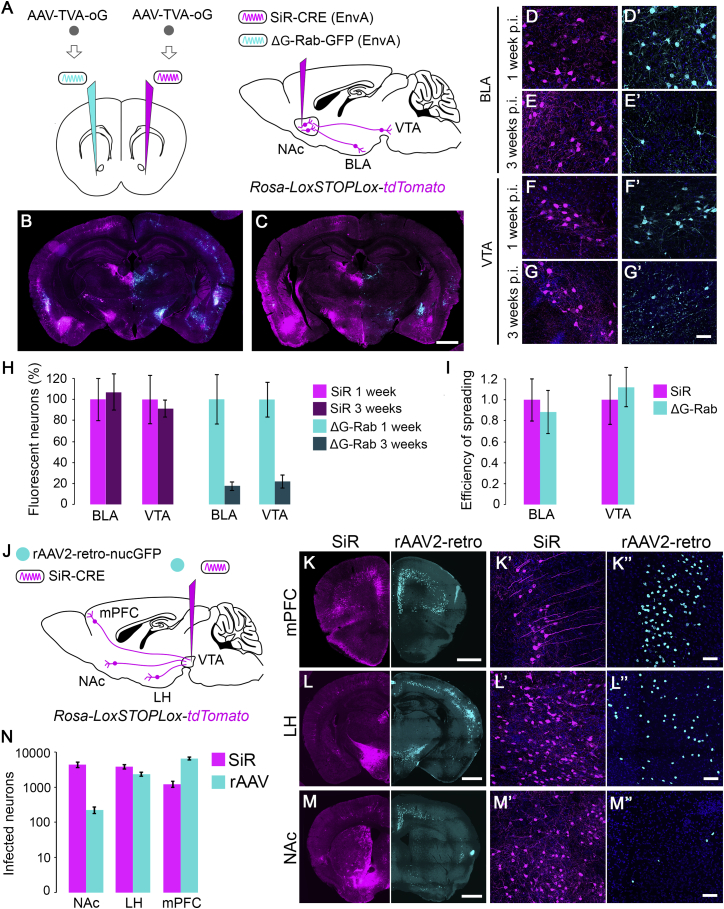
SiR Transsynaptic and Intraneuronal Retrograde Spread (A) Diagram of the injection procedure for the transsynaptic spread from the nucleus accumbens (NAc). (B and C) Spreading at 1 week (B) and 3 weeks p.i. (C) of B19 ΔG-rabies (cyan) and SiR (magenta). Scale bar, 1,000 μm. (D–E′) Transsynaptically labeled neurons in basolateral amygdala (BLA) at 1 week (D and D′) and 3 weeks p.i. (E and E′) with SiR (D and E, magenta) and ΔG-rabies (D′ and E ′, cyan). Scale bar, 50 μm. (F–G′) Transsynaptically labeled neurons in ventral tegmental area (VTA) at 1 week (F and F′) and 3 weeks p.i. (G and G′) with SiR (D and E, magenta) and ΔG-rabies (D′ and E′, cyan). (H) Number of SiR or ΔG-rabies positive neurons at 1 and 3 weeks p.i. in BLA and VTA normalized to 1 week time points (mean ± SEM, n = 3 animals per time point). (I) Efficiency of spreading at 1 week p.i. as number of inputs normalized to number of starting cells, and of SiR and ΔG-rabies in BLA and VTA (SiR = 1, mean ± SEM, n = 3 animals). (J) Scheme of retrograde intraneuronal spreading from VTA. (K–M″) Retrogradely labeled neurons with SiR and rAAV2-retro (SiR, magenta; rAAV2-retro, cyan) in medial prefrontal cortex (mPFC) (K–K″), lateral hypothalamus (LH) (L–L″), and NAc (M–M″). (N) Number of SiR- and rAAV2-retro-infected neurons in NAc, LH, and PFC (mean ± SEM, n = 3 animals) Scale bars, 1,000 μm (K–M); 50 μm (K′, K″, L′, L″, M′, and M″). See also Figure S6.

For its use in long-term functional investigations of neural networks, it is key that the SiR retains the ability to spread transsynaptically. Therefore, we assessed the spreading capabilities of SiR and compared it to the canonical B19 ΔG-rabies in both cortical and subcortical circuits. In order to test this, we first injected the nucleus accumbens (NAc) bilaterally with an AAV expressing TVA and the newly developed optimized rabies glycoprotein (oG) (Figure 4A), which has been shown to enhance rabies virus spreading (Kim et al., 2016). The expression of TVA permits selective infection of the starting cells in the NAc by an EnvA pseudotyped SiR, while the expression of oG allows its transsynaptic retrograde spread (Kim et al., 2016, Wickersham et al., 2007b). Then, we re-targeted the NAc in the two hemispheres with either SiR or ΔG-rabies EnvA-pseudotyped viruses. In agreement with the known connectivity of this area (Russo and Nestler, 2013), we identified transsynaptically labeled neurons in various cortical and subcortical regions, including the basolateral amygdala (BLA) and ventral tegmental area (VTA). We focused on these two areas to quantify spreading efficiency. At 1 week p.i., we observed no significant difference in the spreading capabilities between SiR and the canonical B19 ΔG-rabies (Figures 4A, 4B, 4D, 4D′, 4F, 4F′, and 4I; one-way ANOVA, F = 0.03, p = 0.96), indicating that the self-inactivating nature of the SiR does not affect its ability to spread efficiently. Moreover, as expected, by 3 weeks p.i., we observed a decrease in the number of transsynaptically labeled neurons upon B19 ΔG-rabies infection due to neuronal loss, while no changes upon SiR infection were detected (Figures 4C, 4E, 4E′, and 4G–4H; B19 ΔG-rabies, one-way ANOVA, F = 43, p = 6 × 10^−5^; SiR, one-way ANOVA, F = 0.03, p = 0.96).

**Figure S6:**
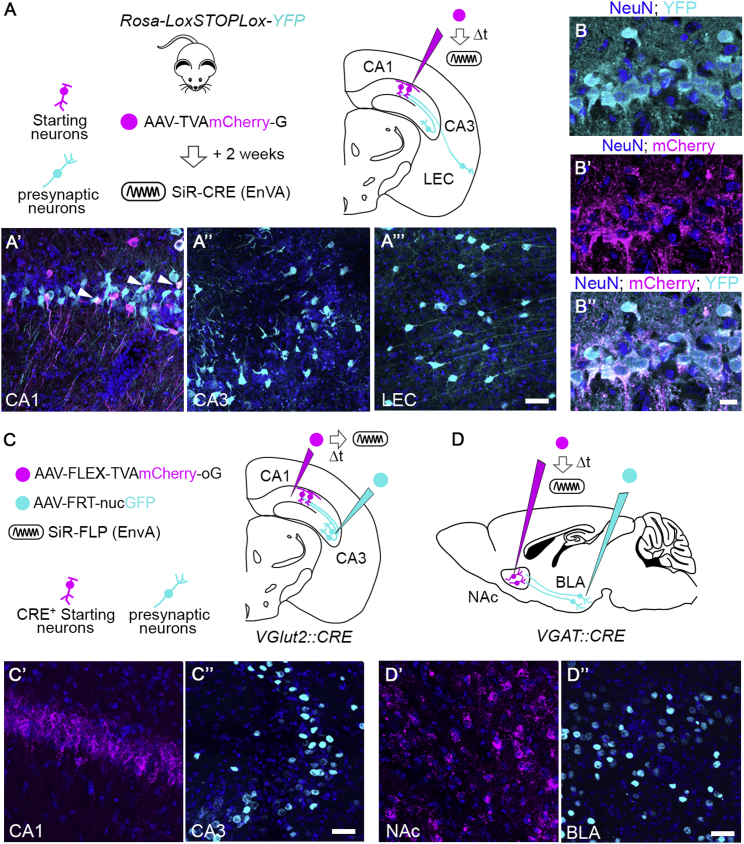
SiR Transsynaptic Spread from Cell-Type-Specific Neurons, Related to Figure 4 (A) AAV^TVAmCherry-G^ was injected in CA1 of *Rosa-LoxP-STOP-LoxP-YFP* mice and the TVA expressing neurons were specifically targeted with an EnvA pseudotyped SiR^CRE^ 2 weeks later. (A’) In the site of injection YFP^ON^/mCherry^ON^ starting neurons are detected (arrowheads) and (A’’-A’’’) the transsynaptic jump of SiR virus permanently labeled neurons in CA3 and lateral enthorinal cortex (LEC) with YFP expression. Scale bar: 25 μm. (B-B’’) Confocal images of CA1 pyramidal neurons infected with AAV^TVAmCherry-G^ and SiR^CRE^ at 3 weeks p.i. Scale bar: 10 μm. (C) AAV-FLEX^TVAmCherry-oG^ was injected in CA1 and AAV-FRT^nucGFP^ in CA3 of *VGlut2::CRE* mice. Two weeks later CRE positive TVA expressing neurons were specifically targeted with an EnvA pseudotyped SiR^FLP^. (C’) Confocal images of excitatory starting neurons in CA1 and (C’’) GFP^ON^ neurons in CA3 activated by SiR^FLP^ transsynaptic spreading. Scale bar: 25 μm (D) AAV-FLEX^TVAmCherry-oG^ was injected in NAc and AAV-FRT^nucGFP^ in BLA of *VGAT::CRE* mice. Two weeks later CRE positive TVA expressing neurons were specifically targeted with an EnvA pseudotyped SiR^FLP^. (D’) Confocal images of VGAT positive starting neurons in NAC and of transsynaptically traced GFP positive neurons in BLA (D’’). Scale bar: 25 μm.

Furthermore, we tested the transsynaptic spreading ability of SiR also in hippocampal circuits by targeting the pyramidal layer of CA1 of *Rosa-LoxP-STOP-LoxP-YFP* mice. As expected, we identified neurons labeled by the SiR in the pyramidal layer of CA3 (Figures S6A and S6A″), indicating specific transsynaptic spreading. Presynaptic neurons were also identified in the lateral entorhinal cortex (LEC) as evidenced by YFP expression (Figure S6A″′). Importantly, SiR-infected starting cells in CA1 expressing TVA and G remained viable throughout the infection period (Figures S6B–S6B″). Thus, efficient transsynaptic spreading was compatible with long-term survival of the synaptically connected neurons.

The use of CRE recombinase in the SiR limits its application in combination with available mouse CRE lines. This is a limiting factor when trying to initiate the transsynaptic spreading from genetically defined neuronal populations. To overcome this shortcoming, we generated an SiR encoding for a FLP recombinase and tested its efficiency. We specifically targeted CA1 pyramidal neurons by injecting a CRE-dependent AAV expressing TVA and oG (AAV-FLEX^TVA-oG^) in CA1 of *VGlut2::CRE* mice, in which the CRE recombinase is expressed in excitatory neurons (Figure S6C). At the same time, we targeted the presynaptic area CA3 with a FLP-dependent AAV encoding a nuclear-tagged GFP (AAV-FRT^nucGFP^) (Figure S6C). The AAV-FRT^nucGFP^ virus drives GFP expression following a FLP-dependent recombination event. We then injected SiR^FLP^ (EnvA) in CA1 initiating the transsynaptic spreading selectively from TVA-expressing excitatory neurons (Figures S6C and S6C′). Transsynaptically labeled neurons can be observed in the CA3 presynaptic target area (Figure S6C″), proving the viability of the approach.

In order to confirm the general validity of this method, we decided to use a distinct transgenic line to start the transsynaptic spreading also from inhibitory neurons.

We first injected AAV-FLEX^TVA-oG^ in the NAc of *VGAT::CRE* mice to selectively target the inhibitory neuronal population in this area. We then injected the AAV-FRT^nucGFP^ in a known presynaptic area, the BLA (Figure S6D). Then, we specifically targeted TVA-positive neurons with SiR^FLP^ (EnvA), thus initiating the transsynaptic spread selectively from NAc inhibitory neurons (Figures S6D and S6D′). This led to efficient FLP-dependent transsynaptic labeling of neurons in BLA (Figure S6D″). Overall, these results confirm the efficiency of a CRE- and FLP-dependent combinatorial approach based on the use of SiR^FLP^ in conjunction with available CRE mouse lines, expanding the range of use of the SiR.

### Use of SiR as Retrograde Non-transsynaptic Tracer

Given the paucity of highly efficient and selective axonal retrograde viral tracers, we took advantage of the high affinity of the rabies virus for axon terminals and assessed whether the SiR can be used also as retrograde intraneuronal (non-transsynaptic) tracer. To this aim, we pseudotyped the SiR with oG (Kim et al., 2016). Using this approach, we mapped the synaptic inputs to the VTA with SiR (oG) comparing its non-transsynaptic retrograde spreading efficiency with one of the most efficient retrograde viruses available to date, the rAAV2-retro (Tervo et al., 2016). We focused our analysis on three known presynaptic areas to VTA: the medial prefrontal cortex (mPFC), the NAc, and the lateral hypothalamus (LH) (Figure 4J) (Watabe-Uchida et al., 2012). Overall, spreading efficiency is broadly comparable between SiR and rAAV2-retro (Figure 4N). However, SiR was more efficient in labeling subcortical structures such as LH and NAc (LH, p = 0.18; NAc, p = 0.03; Figures 4L–4L″, 4M–4M″, and 4N), with more than one order of magnitude of difference in the case of the NAc to VTA circuit. On the other hand, the rAAV2-retro turned out to be more efficient in labeling cortical targets such as the mPFC (p = 0.05, Figures 4K–4K″ and 4N).

Altogether, these data indicate that SiR also functions as a highly effective retrograde axonal (non-transsynaptic) tracer.

### Unaffected Neuronal and Synaptic Functions on SiR Infection

**Figure 5:**
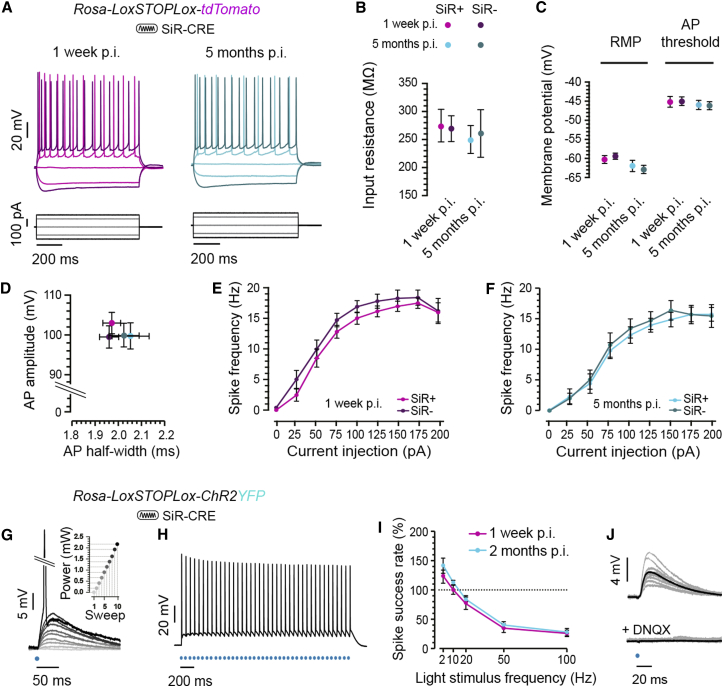
SiR Infection Has No Long-Term Impact on Neuronal Physiology (A) Membrane potential response to steps of positive and negative current of a CA1 pyramidal neuron 1 week and 5 months p.i. of SiR^CRE^ in *Rosa-LoxP-STOP-LoxP-tdTomato* mice. (B–F) (B) Input resistance, (C) resting membrane potential (RMP) and action potential (AP) threshold, (D) AP amplitude and width, and (E and F) firing frequency at increasing steps of positive current for SiR^+^ neurons expressing tdTomato at 1 week p.i. (light magenta, n = 14 neurons) and 5 months p.i. (light cyan, n = 10). SiR- neighboring neurons were also recorded: tdTomato- neurons at 1 week p.i. (dark magenta, n = 14) and 5 months p.i. (dark cyan, n = 9; mean ± SEM). (G) *Rosa-LoxP-STOP-LoxP-ChR2YFP* mice were injected with SiR^CRE^. Membrane potential response to a 0.2 ms blue light pulse of increasing intensity (by 1% in each sweep until spiking; 0% lighter gray to 9% black) was assessed in SiR^CRE^-infected CA1 neuron expressing ChR2 (1 week p.i.). Inset: LED power delivered for each sweep. (H) Membrane potential response to 40 1.5-ms long light pulses at 20 Hz (2.17 mW). (I) Action potentials success rate following 40 light stimulations at increasing frequencies, 1 week (magenta, n = 17) and 2 months (cyan, n = 19) p.i. (J) Light-evoked EPSPs recorded in non-ChR2-expressing neurons blocked by DNQX (20 μM). Average traces for both conditions are shown in black. See also Table S2.

While the absence of cytotoxicity and the maintenance of transsynaptic spreading ability of the SiR is encouraging for its use in the functional manipulation of neural networks, we also wanted to make sure that SiR has no long-term effects on neuronal physiology. In order to do so, we injected SiR^CRE^ in the pyramidal layer of CA1 in *Rosa-LoxP-STOP-LoxP-tdTomato* mice. We then compared the electrophysiological properties of infected and non-infected neurons at 1 week and 5 months following SiR infection. Tomato^ON^ neurons in CA1 were recorded in whole-cell patch-clamp mode. All pyramidal CA1 neurons recorded showed regular spiking profiles (Figure 5A) with no significant difference among all tested conditions and time points in the input resistance, resting membrane potential, spike threshold, action potential amplitude and half-width, and instantaneous firing frequency (Figures 5B–5F; Table S2). Furthermore, when SiR^CRE^ was injected in *Rosa-LoxP-STOP-LoxP-ChR2YFP* mice, ChR2 was successfully expressed in transduced neurons (Figures 5G–5J). Brief light pulses (0.5–2 ms) lead to ChR2 direct activation in transduced neurons and triggering of action potentials with short latency (5.4 ± 0.6 ms spike-peak latency, Figure 5G–5H). Infected neurons could be activated at various frequencies with similar reliability at both 1 week and 2 months p.i. (Figure 5I). A key finding was that light activation of SiR^CRE^-infected neurons elicited DNQX-sensitive excitatory postsynaptic potentials (EPSPs) (latency 6.8 ± 0.4 ms) in non-infected neurons at 2 months p.i. (Figure 5J), indicating persistence of functional connectivity and no adverse effects on synaptic function.

### Investigating Network Dynamics with SiR

**Figure 6:**
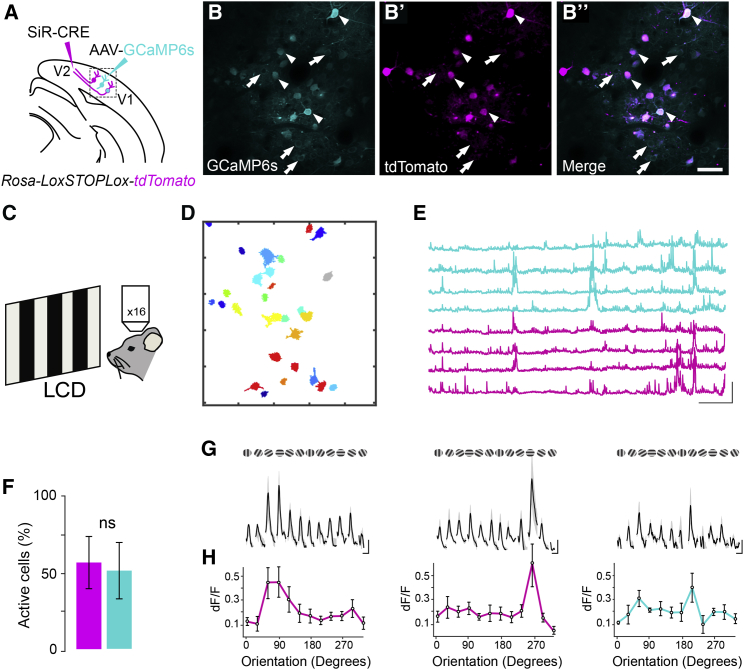
Unaltered Orientation Tuning Responses of SiR-Traced V1 Neurons (A) Schematic of SiR^CRE^ and AAV^GCaMP6s^ injection in *Rosa-LoxP-STOP-LoxP-tdTomato* mice in V2 and V1, respectively. (B–B″) Two-photon maximal projection of V1 neurons after SiR^CRE^ injection. In cyan neurons expressing GCaMP6s (B), in magenta neurons expressing tdTomato (B′), and in the merge neurons expressing both (B″, merge). Arrows and arrowheads highlight representative GCaMP6s or GCaMP6s-tdTomato expressing neurons, respectively. Scale bar, 50 μm. (C) Schematic of visual stimulation set up. (D) Outline of the active ROIs from the same field of view shown in (B). (E) Representative Ca^2+^ traces of GCaMP6s (cyan) and GCaMP6s-tdTomato (magenta) neurons. Scale bars, 200 s, 20% dF/F_0_. (F) Mean percentage of active neurons after 4 weeks from SiR injection (n = 163 GCaMP6s neurons [cyan], n = 78 GCaMP6s-tdTomato neurons [magenta]; mean ± SEM). (G) Changes in fluorescence over time reflecting visual responses to drifting gratings at the preferred direction of each neuron. (H) Example of tuning curve of V1-infected neurons (mean ± SEM). Scale bars, 5 s, 10% dF/F_0_. See also Figure S7 and Movie S1.

**Figure S7:**
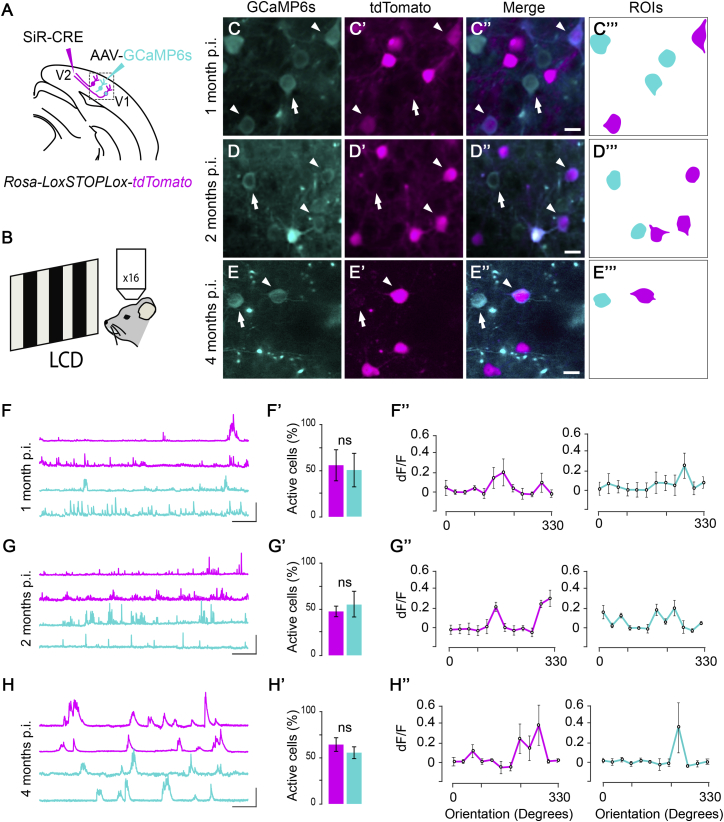
The Orientation Tuning Responses of SiR traced V1 neurons are preserved up to 4 months from the injection, Related to Figure 6 (A) Schematic of SiR^CRE^ and AAV^GCaMP6s^ injection in *Rosa-LoxP-STOP-LoxP-tdTomato* mice in V2 and V1 respectively. (B) Schematic of visual stimulation set up. (C-E’’’) Two-photon maximal projection of V1 neurons 1, 2, or 4 months after SiR^CRE^ injection. In cyan neurons expressing GCaMP6s (C, D, E), in magenta neurons expressing tdTomato (C’, D’, E’) and in the merge neurons expressing both (C’’, D’’, E’’ merge). Arrows and arrowheads highlight representative GCaMP6s (cyan) or GCaMP6s-tdTomato (magenta) expressing neurons respectively. Scale bar: 20 μm. (C’’’, D’’’, E’’’) Outline of the active ROIs from the same field of view showed in panel C, D and E. (F, G, H) Representative Ca^2+^ traces of GCaMP6s (cyan) and GCaMP6s-tdTomato (magenta) neurons. Scale bars: 100 s, 20% dF/F_0_. (F’, G’, H’) Mean percentage of active neurons after 1, 2 or 4 months from SiR injection (n = 241, 156, 231 respectively, mean ± SEM). (F’’, G’’, H’’) Example of tuning curve of V1 infected neurons after 1, 2 or 4 months (GCaMP6s neurons (cyan) and GCaMP6s-tdTomato neurons (magenta); dF/F_0_ mean ± SEM).

The absence of cytotoxicity and unaltered electrophysiological responses support the use of SiR for long-term circuit manipulations. The presence of functional connectivity between SiR-infected neurons and no adverse effects on synaptic properties also indicate that network function is likely to be preserved following SiR infection. In order to test whether network-dependent computations are also preserved and whether SiR can be used to monitor neural activity in vivo, we looked at a prototypical example of network-dependent computation, orientation tuning in visual cortex. We first targeted V1 neurons projecting to V2 (V1 ^> V2^) by injecting oG pseudotyped SiR^CRE^ in the V2 area of *Rosa-LoxP-STOP-LoxP-tdTomato* mice. At the same time, we injected an AAV^GCaMP6s^ in the ipsilateral V1 (Figure 6A). Retrograde spreading of SiR^CRE^ from V2 induced recombination of the Rosa locus permanently labeling V1 ^> V2^ neurons (Figures 6B–6B″; Movie S1). We then monitored the Ca^2+^ dynamics of SiR-infected V1 ^> V2^ neurons in vivo 4 weeks p.i., under a two-photon microscope, while anesthetized animals were exposed to moving gratings of different orientations across the visual field (Figure 6C) (Benucci et al., 2009). Infected V1 ^> V2^ neurons showed significant increases in fluorescence at particular grating orientations resulting in a cellular tuning curve showing direction or orientation selectivity (Figures 6G and 6H). Notably, recorded Ca^2+^ responses, as well as the percentage of active neurons, were similar between SiR-traced neurons (GCaMP6s^ON^-tdTomato^ON^) and neighboring non-SiR V1 neurons (GCaMP6s^ON^-tdTomato^OFF^) (Figures 6E and 6F). Furthermore, to exclude the possibility of long-term deleterious effects on circuit function following SiR infection, we repeated functional imaging experiments at 2 and 4 months p.i. (Figure S7). In both cases, we found no significant difference in percentage of active neurons or stimulus-driven responses between GCaMP6s^ON^-tdTomato^ON^ and GCaMP6s^ON^-tdTomato^OFF^ neurons (Figures S7F–S7H″). These data indicate that SiR-traced networks preserve unaltered computational properties and that SiR can be used in combination with GCaMP6s to monitor the Ca^2+^ dynamic with no upper bounds to the temporal window for the optical investigation.

## Discussion

The development of monosynaptically restricted rabies viruses has had a transformative effect on the study of neural circuits (Miyamichi et al., 2011, Stepien et al., 2010, Tripodi et al., 2011, Wickersham et al., 2007b). Until now, however, the cellular cytotoxicity that accompanies rabies virus infection effectively limited the temporal window for its use for the functional interrogation of neural circuits. The induced cytotoxicity is linked to the transcriptional activity of the virus. Replicative and transcriptionally competent rabies viruses hijack the cellular translational machinery to sustain their replication at the expense of endogenous cellular translation (Komarova et al., 2007). This affects cellular function, cellular transcriptional profile, and eventually, neuronal function and survival. Therefore, any replicative competent rabies virus, including less cytotoxic strains (Reardon et al., 2016), will eventually compromise cellular physiology within a particular time frame. In order to gain life-long genetic and functional access to network elements defined topologically by rabies-dependent gene expression and in the absence of any adverse cytotoxic effects, we developed a SiR that transcriptionally disappears from infected neurons shortly after the primary infection but has sufficient transcriptional activity in those early stages to genetically tag infected neurons for the long-term.

In line with the complete transcriptional silencing of the virus, we observed no changes in the electrophysiological signature between infected and uninfected neurons 1 week post infection, when viral transcription is dampened but still present, and 5 months after infection, a time point at which the SiR has switched OFF but that is well beyond the survival window of neurons infected with canonical B19 ΔG-rabies (Wickersham et al., 2007a). Moreover, we showed that SiR virus can be employed in long-term studies using optogenetics with no reduction in the reliability of optical stimulation and, most importantly, maintaining the synaptic function and integrity that is key for physiological and behavioral studies. By using SiR to monitor calcium dynamics in vivo, we show that circuit-dependent responses of infected neurons, such as visual tuning to moving stimuli, remain unaffected after viral infection.

**Figure 7:**
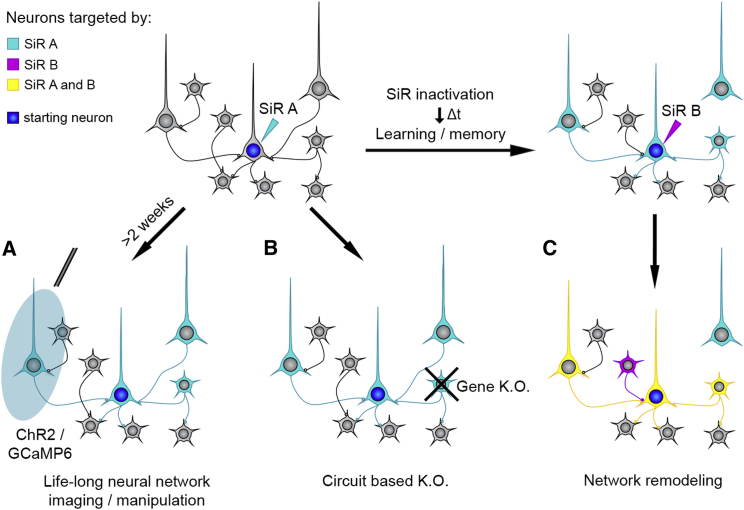
SiR Applications Address the contribution of topologically defined neurons to behaviors (A); manipulate gene expression of a specific network’s node (B); and investigate network remodeling following memory formation and learning (C) by retargeting the network with an SiR expressing a different label (cyan, connections lost; magenta, connections gained; yellow, connections retained).

The unique features of the SiR make it the ideal tool for long-term functional and genetic modification of neural networks. Such interventions may include the functional manipulation of neural circuits with light-activated opsins or chemically gated channels as well as the study of long-term network activity using genetically encoded calcium indicators (Emiliani et al., 2015, Luo et al., 2008, Packer et al., 2013, Packer et al., 2015) (Figure 7A). With the canonical B19 ΔG-rabies, the safe temporal window that ensures near-physiological neuronal responses and minimal neuronal loss has been reported to be between 5–15 days from the infection, with slight differences between reports, possibly reflecting different sensitivity of distinct neuronal populations (Kiritani et al., 2012, Namburi et al., 2015, Tian et al., 2016, Wertz et al., 2015). The use of the recently introduced ΔG-rabies strain variant CVS-N2c^ΔG^ has been reported to push the useful temporal window for imaging further up to 17 days post infection (Reardon et al., 2016). With SiR, traced neurons remain healthy indefinitely, and there are no upper bounds to the temporal window for the optical or functional investigation of neural circuits in physiological conditions except for those imposed by the imaging approach itself and the expressed transgene of choice.

SiR also makes it possible to knockout genes of interest in a circuit-specific manner via genomic recombination of a conditional cassette, for the first time without any associated neuronal loss or temporal constraints (Figure 7B). In addition, the uniquely transient transcriptional nature of SiR combined with permanent CRE-dependent tracing offers the possibility to trace recurrently the circuit upstream of a defined entry node over time, leaving a permanent snapshot of the network topology at each iteration (Figure 7C). This feature may be exploited to follow circuit remodeling after physiological or pathological structural plasticity, such as during development, upon learning or following traumatic brain injuries.

Switching OFF viral transcription is not only instrumental to abolish cell death and preserve neuronal function, but it also eliminates the problem of the rabies-mediated inhibitory effect on the cellular translational machinery (Komarova et al., 2007). Moreover, a further advantage of the self-inactivating nature of the virus is that it may allow for lowering the biosafety requirement for its production. We show that upon infection in vivo, there are no detectable viral particles in the injected brain tissue after 2 weeks from the primary infection, which may lead to downgrading SiR safety level.

One caveat to consider when using SiR is that, while our design produces a neurotropic virus that has no adverse effects on neuronal function and survival, this is at the cost of switching OFF its own transcription. Therefore, the delivery of transgenes of interest must be achieved in a combinatorial manner by making use either of floxed viral cassettes (i.e., AAVs) or of transgenic conditional mouse lines. Notably, we successfully implemented both strategies in this study.

Our findings establish SiR as a tool that provides functional and genetic access to traced network elements safely in vivo for the lifetime of the animal, with no cytotoxic effects, no changes in the intrinsic electrophysiological properties of the infected neurons, and no adverse effects on network function and network-dependent computations, opening new horizons in the functional investigation of neural circuits.

## STAR★Methods

### Key Resources Table

**Table:** 

REAGENT or RESOURCE	SOURCE	IDENTIFIER
**Antibodies**		

Chicken polyclonal anti-GFP	Thermo Fisher Scientific	Cat#A10262; RRID: AB_2534023
Rabbit polyclonal anti-RFP	Rockland	Cat#600-401-379; RRID: AB_2209751
Mouse monoclonal anti-V5	Sigma-Aldrich	Cat#V8012; RRID: AB_261888
Rabbit polyclonal anti-cleaved caspase-3 (Asp175)	NEB	Cat#9661; RRID: AB_2341188
Donkey polyclonal anti-rabbit Cy3	Jackson ImmunoResearch	Cat#711-165-152; RRID: AB_2307443
Donkey polyclonal anti-chicken alexa 488	Jackson ImmunoResearch	Cat#703-545-155; RRID: AB_2340375
Goat polyclonal anti-Mouse IgG (H+L) HRP	Thermo Fisher Scientific	Cat#32430; RRID: AB_1185566

**Chemicals, Peptides, and Recombinant Proteins**		

RabiesΔG-mCherry	This paper	N/A
RabiesΔG-_N-ter_TAGs-mCherry	This paper	N/A
RabiesΔG-_N-ter_TAGs-N^PEST^-mCherry	This paper	N/A
RabiesΔG-_N-ter_TAGs-M^PEST^- mCherry	This paper	N/A
RabiesΔG-_N-ter_TAGs-P^PEST^- mCherry	This paper	N/A
RabiesΔG-_N-ter_TAGs-L^PEST^- mCherry	This paper	N/A
RabiesΔG-N^PEST^-mCherry (SiR-mCherry)	This paper	N/A
RabiesΔG-_N-ter_TAGs-(P+L)^PEST^- mCherry	This paper	N/A
RabiesΔG-(P+L+N)^PEST^- mCherry	This paper	N/A
RabiesΔG-N^PEST^ -iCRE-2A-mCherryPEST (SiR-CRE-mCherry)	This paper	N/A
RabiesΔG-N^PEST^-_C_TEVp-FKBP-2A-FRB-_N_TEVp-iCRE	This paper	N/A
RabiesΔG-N^PEST^ -FLPo (SiR-FLP)	This paper	N/A
Lenti-_H2B_GFP-2A-GlySAD	This paper	N/A
Lenti-puro-2A-TEVp	This paper	N/A
Lenti-GFP	This paper	N/A
AAV2/9-CMV-TVAmCherry-2A-Gly	This paper	N/A
AAV2/9-TRE_tight_-TEVp-CMV-rTTA	This paper	N/A
AAV2/9-CMV-FRT-_H2B_GFP	This paper	N/A
AAV2/9-CMV-FLEX-TVAmCherry-2A-oG	This paper	N/A
AAV2/9-CAG-GCaMP6s	Penn Vectore Core	N/A
Puromycin dihydrochloride	Thermo Fisher Scientific	Cat#A1113802
Doxycycline hydrochloride	Santa-Cruz Biotech	Cat#00929-47-3
MG-132	Sigma-Aldrich	Cat#M7449
Polyethyleneimine	Polysciences	Cat#24765
Rapamycin	LKT Laboratories	Cat#R0161
DNQX (6,7-Dinitroquinoxaline-2,3-dione)	Tocris Bioscience	Cat#0189

**Critical Commercial Assays**		

Plasmid plus Maxi kit	QIAGEN	Cat#12943
Gibson Assembly master mix	NEB	Cat#E2611S
Q5 hot start DNA polymerase	NEB	Cat#M0493S
SuperScript IV Reverse Transcriptase	Thermo Fisher Scientific	Cat#18090050
Rotor-Gene SYBR Green PCR Kit	QIAGEN	Cat#204074
RNeasy Mini kit	QIAGEN	Cat#74104

**Experimental Models: Cell Lines**		

HEK-GG	This paper	N/A
HEK-TGG	This paper	N/A
BHK-TGoG	This paper	N/A
BHK-T-EnVA	This paper	N/A

**Experimental Models: Organisms/Strains**		

Mouse: C57BL/6J	Jackson Laboratory	JAX: 000664
Mouse: Rosa-LoxP-STOP-LoxP-tdtomato: Gt(ROSA)26Sortm14(CAG tdTomato	Jackson Laboratory	JAX: 007914
Mouse: Rosa-LoxP-STOP-LoxP-YFP: Gt(ROSA)26Sor < tm1(EYFP)Cos > )	Jackson Laboratory	JAX: 006148
Mouse: Rosa-LoxP-STOP-LoxP-ChR2-YFP: B6.Cg-Gt(ROSA)26Sortm32(CAG-COP4^∗^H134R/EYFP)Hze/J)	Jackson Laboratory	JAX: 024109
Mouse: VGAT::CRE: Slc32a1tm2(cre)Lowl	Jackson Laboratory	JAX: 016962
Mouse: VGlut2::CRE: Slc17a6tm2(cre)Lowl	Jackson Laboratory	JAX: 016963

**Recombinant DNA**		

pcDNA-B19N	Callaway E., Salk Institute	N/A
pcDNA-B19P	Callaway E., Salk Institute	N/A
pcDNA-B19L	Callaway E., Salk Institute	N/A
pcDNA-B19G	Callaway E., Salk Institute	N/A
pCAG-T7pol	Callaway E., Salk Institute	N/A
pSAD-F3-mCherry	This paper	N/A
pSAD-F3-_N-ter_TAGs-mCherry	This paper	N/A
pSAD-F3-_N-ter_TAGs-N^PEST^-mCherry	This paper	N/A
pSAD-F3-_N-ter_TAGs-M^PEST^- mCherry	This paper	N/A
pSAD-F3-_N-ter_TAGs-P^PEST^- mCherry	This paper	N/A
pSAD-F3-_N-ter_TAGs-L^PEST^- mCherry	This paper	N/A
pSAD-F3-N^PEST^-mCherry (SiR-mCherry)	This paper	N/A
pSAD-F3-_N-ter_TAGs-(P+L)^PEST^- mCherry	This paper	N/A
pSAD-F3-(P+L+N)^PEST^- mCherry	This paper	N/A
pSAD-F3-N^PEST^ -iCRE-2A-mCherryPEST (SiR-CRE-mCherry)	This paper	N/A
pSAD-F3-N^PEST^-_C_TEVp-FKBP-2A-FRB-_N_TEVp-iCRE	This paper	N/A
pSAD-F3-N^PEST^ -FLPo (SiR-FLP)	This paper	N/A
pLenti-_H2B_GFP-2A-GlySAD	This paper	N/A
pLenti-puro-2A-TEVp	This paper	N/A
pLenti-GFP	This paper	N/A
pAAV2/9-CMV-TVAmCherry-2A-Gly	This paper	N/A
pAAV2/9-CMV-FRT-_H2B_GFP	This paper	N/A
pAAV2/9-CMV-FLEX-TVAmCherry-2A-oG	This paper	N/A
pAAV2/9-TRE_tight_-TEVp-CMV-rtTA	This paper	N/A

**Software and Algorithms**		

ImageJ (Fiji 1.48)	Fiji	https://fiji.sc/
NIS-Elements HCA 4.30	Nikon	https://www.nikoninstruments.com/en_GB/Products/Software/NIS-Elements-HC
MATLAB	Mathworks	https://www.mathworks.com/products/matlab/
Python 2.7 (Anaconda 4.2.0 distribution)	Continuum Analytics	https://www.continuum.io
R	The R project	https://www.r-project.org/

**Other**		

### Contact for Reagents and Resource Sharing

Further information and requests for resources and reagents should be directed to and will be fulfilled by the Lead Contact, Marco Tripodi (mtripodi@mrc-lmb.cam.ac.uk).

### Experimental Model and Subject Details

#### Animal strains

Male mice aged between 8 and 12 weeks from of the following lines were used: C57BL/6 wild-type (WT), Rosa-LoxP-STOP-LoxP-tdTomato (Jackson: Gt(ROSA)26Sortm14(CAG tdTomato), Rosa-LoxP-STOP-LoxP-YFP (Jackson: Gt(ROSA)26Sor < tm1(EYFP)Cos > ), Rosa-LoxP-STOP-LoxP-ChR2-YFP (Jackson: B6.Cg-Gt(ROSA)26Sortm32(CAG-COP4^∗^H134R/EYFP)Hze/J), VGAT::CRE (Jackson: Slc32a1tm2(cre)Lowl), VGlut2::CRE (Jackson: Slc17a6tm2(cre)Lowl). All transgenic mice were isogenic in a C57BL/6 background, maintained in pathogen and opportunistic agents-free conditions and monitored quarterly. All procedures were conducted in accordance with the UK Animals (Scientific procedures) Act 1986 and European Community Council Directive on Animal Care. Animals were group-housed in a 12 hr light/dark cycle with food and water ad libitum.

#### Cell lines

HEK293T cells and BHK-21 were purchased from ATTC. All cell lines were maintained in Dulbecco’s modified Eagle’s medium (DMEM, GIBCO) supplemented with 10% fetal bovine serum (FBS, GIBCO) and 1% Penicillin/Streptomycin (GIBCO) unless otherwise stated. HEK293T packaging cells expressing Rabies glycoprotein (HEK-GG) were generated by lentivirus infection with Lenti-_H2B_GFP-2A-GlySAD (Table S1) and after 3 passages GFP expressing cells were selected by fluorescent activated cell sorting (FACS). HEK293T packaging cells expressing Rabies glycoprotein and TEV protease (HEK-TGG) were generated from HEK-GG by lentivirus infection with Lenti-puro-2A-TEVp and selected, after 3 passages, with 1 μg/mL of puromycin added to the media for 1 week.

BHK-21 packaging cells for pseudotyping Rabies virus with optimized G (BHK-TGoG) were generated with the same procedure as the HEK-TGG by the infection first with Lenti-_H2B_GFP-2A-oG and subsequently with Lenti-puro-2A-TEVp. BHK packaging cells for pseudotyping Rabies virus with EnvA receptor (BHK-T-EnvA) were obtained infecting BHK-EnvA with Lenti-puro-2A-TEVp and selecting with puromycin.

### Method Details

#### First generation ΔG-N^PEST^Rabies. In vitro and in vivo tests of cytotoxicity

In order to obtain conditional regulation of viral protein stability, in addition to the C-Terminal PEST domain (see main text), a SPLIT-TEVp cassette (Gray et al., 2010) was cloned in the ΔG-Rabies genome. A tag (myc, FLAG or V5) was also fused to the N-terminal of each viral protein. The SPLIT-TEVp dimeric protease is only active in presence of rapamycin and could potentially provide a tool for the exogenous regulation of viral protein stability during production and in vivo. We first tested the capability of the SPLIT-TEVp expressed by plasmid to cleave a TEVp activity reporter in HEK293T cells (Figure S2B), then we tested the ability of the virally expressed cassette to cleave the TEVp activity reporter (Figure S2C).

In order to probe the effect of protein destabilization on neuronal survival, we infected human Embryonic Stem cells (hESCs) derived neurons with ΔG-N^PEST^Rabies^SPLIT-TEVp-mCherry^. We performed a longitudinal study of the survival of infected neurons and compared survival rate to a control ΔG-Rabies. Neurons were infected and imaged longitudinally at 4, 10 and 16 days to evaluate the cell death (Figures S3A–S3C″). Lentivirus expressing GFP was used to normalize infection rates in order to account for cell death due to the prolonged manipulation and repeated over-night imaging sessions. Only 26% ± 4% of control ΔG-Rabies infected neurons were still detectable at 16 days post-infection (N = 3, n = 781 for each condition; Figures S3C–S3C″ and S3D). On the contrary, the ΔG-N^PEST^Rabies^SPLIT-TEVp-mCherry^ virus showed no significant cell loss after 16 days (94% ± 6%, N = 3, n = 917 for each condition, Figures S3B–S3B″ and S3D) and a significant increase in cell survival compared to ΔG-Rabies control (p = 3.2x10^−5^; paired two-tailed Student’s t test).

To understand if the reduced cytotoxicity of ΔG-N^PEST^Rabies^SPLIT-TEVp-mCherry^ is associated with a reduction of the viral transcription, we monitored the intensity of the reporter expressed in neurons infected with the control ΔG-Rabies and the ΔG-N^PEST^Rabies^SPLIT-TEVp-mCherry^ viruses which share the same expression cassette (Figure S3E). Over time the mean mCherry signal of ΔG-N^PEST^Rabies^SPLIT-TEVp-mCherry^ infected cells resulted to be significantly lower than controls (mCherry intensity at 10 days, ΔG-Rabies 138% ± 3%, ΔG-N^PEST^Rabies^SPLIT-TEVp-mCherry^87% ± 7%, p = 0.01; N = 3, n = 584 for each condition; paired two-tailed Student’s t test).

We then tested the performance of the ΔG-N^PEST^Rabies^SPLIT-TEVp-mCherry^ virus in vivo.

We replaced the mCherry gene in the ΔG-N^PEST^Rabies^SPLIT-TEVp-mCherry^ virus with the CRE recombinase (ΔG-N^PEST^Rabies^SPLIT-TEVp-CRE^). This ensures that infected neurons can be permanently labeled after a complete transcriptional shut down of the virus, allowing for the discrimination between viral silencing and cell death. We injected ΔG-N^PEST^Rabies^SPLIT-TEVp-CRE^ in CA1 of *Rosa-LoxP-STOP-LoxP-tdTomato* reporter mouse line in CA1 in the hippocampus (Figure S3F). We observed a significant increase in neuronal survival upon ΔG-N^PEST^Rabies^SPLIT-TEVp-CRE^ infection compared to that observed upon infection with control ΔG Rabies (25% ± 2%, at 2 weeks for ΔG-N^PEST^Rabies^SPLIT-TEVp-CRE^ and 8% ± 3%, at 2 weeks for ΔG Rabies p = 7x10^−3^, Figures S3G–S3I). However prominent neuronal loss was still present upon ΔG-N^PEST^Rabies^SPLIT-TEVp-CRE^ infection (76% ± 3%, at 3 weeks, p = 9x10^−4^, Figures S3H–S3H″ and S3I).

The residual cytotoxicity of ΔG-N^PEST^Rabies^SPLIT-TEVp-CRE^ might be linked to a constitutive low basal dimerization and activity of the SPLIT-TEVp cassette and can give origin to transcriptionally active viral particles. Consistent with this hypothesis, we observed no significant effect on neuronal survival and mCherry expression levels in presence or absence of rapamycin (mCherry expression 10 days p.i. ΔG-N^PEST^Rabies^SPLIT-TEVp -RAP^ 87% ± 7%, ΔG-N^PEST^Rabies^SPLIT-TEVp +RAP^ 85% ± 6%, p = 0.21; N = 3, n = 793 for each condition; paired two-tailed Student’s t test; Figure S3E) and no significant effects on cells survival were associated with the rapamycin administration in hESCs derived neurons (at 16 days; ΔG-Rabies^+RAP^ 26% ± 4%, ΔG-Rabies^-RAP^ 30% ± 7%, p = 0.69; ΔG-N^PEST^Rabies^SPLIT-TEVp +RAP^ 88% ± 11%, ΔG-N^PEST^Rabies^SPLIT-TEVp -RAP^ 94% ± 6%, p = 0.79; N = 3, n = 833 for each condition; paired two-tailed Student’s t test, Figure S3D). Furthermore, we observed a constitutive low level of TEVp activity in HEK293T cells in absence of Rapamycin (Figure S2B, line 2) indicating a basal level of Rapamycin-independent SPLIT-TEVp dimerization. Overall these results indicate that ΔG-N^PEST^Rabies^SPLIT-TEVp^ has reduced cytotoxicity in hESCs derived neurons and in vivo when compared to ΔG-Rabies. However, it fails to completely switch off following the infection, which leads to significantly delayed, yet still present, neuronal cytotoxicity and neuronal loss. For these reasons we generated a second generation of ΔG-N^PEST^Rabies by removing the leaking SPLIT-TEVp and replacing it with an mCherry-CRE cassette giving origin to a Self inactivating ΔG-Rabies virus (SiR) with the desired ON-OFF and TEVp dependent kinetics (main text).

#### Design and generation of ΔG-Rabies and lentivirus plasmids

All the attenuated Rabies plasmids, listed in Table S1, were generated by Gibson cloning using pSAD-ΔG-F3 plasmid (Osakada et al., 2011) as starting material. Briefly, the Rabies genome was PCR amplified in 2 fragments starting from the protein to be tagged. These fragments were then mixed with the tag and/or PEST domain obtained by oligonucleotides annealing and assembled using Gibson master mix (NEB).

The lentiviral vectors used to generate the packaging cells were derived from a 3^rd^ generation lentivirus transfer vector (gift from Michael Hastings “361 polylinker,” originally pCCL-SIN-18PPT.Pgk.EGFP-WPRE). All the lentiviral vectors were generated by Gibson assembly, opening the backbone by digestion with XbaI and KpnI and PCR amplifying the CMV promoter and the different inserts.

#### Viral Screening and proteasome inhibition

For screening of attenuated ΔG-Rabies viruses, HEK-GG or HEK-TGG cells were co-transfected with rabies genome vector, pcDNA-T7, pcDNA-SADB19N, pcDNA-SADB19P, pcDNA-SADB19L, and pcDNA-SADB19G (Osakada et al., 2011) and maintained at 37°C with 5% CO_2_ humidified atmosphere in DMEM supplemented with 10% FBS (GIBCO) and 100 u/ml Penicillin-Streptomycin. The day after transfection and subsequently every 3 days, cells were washed with PBS, treated with 0.05% trypsin-EDTA and replated in a new dish in a ratio 1:3. After splitting, cells were maintained for one day at 37°C and 5% CO_2_ and then 2 days at 35°C and 3% CO_2_. Every 3 days cells were fixed with 4%PFA and viral spreading was assessed by FACS sorting the cells for mCherry expression.

For testing the proteasome dependence of attenuated ΔG-Rabies virus conditional inactivation, HEK-GG cells were co-transfected and processed as described before for the viral screening. At 3 days post transfection cells were treated with 10-100-250 nM of MG-132 (Sigma) or vehicle (0 nM condition, DMSO) in the medium until the end of the experiment. For the 250 nM MG-132 condition every 48 hr of incubation in DMEM supplemented with MG-132 cells were washed with PBS once and incubated in DMEM 10% FBS for 24 hr.

#### Viral productions

For the recovery of high titer ΔG-Rabies, HEK-GG or HEK-TGG for control or attenuated Rabies respectively, were infected in 10 cm dishes at 70%–80% confluence with 1 mL of viral supernatant obtained as described in the viral screening section. Cells were split the day after infection and maintained for 1 or 2 days at 37°C and 5% CO_2_ checking daily the viral spreading. When 70%–80% of cells expressed the viral marker, the media was replaced with 2% FBS DMEM and maintained for 2 days at 35°C and 3% CO_2_. Then, the viral supernatant was collected, cell debris removed by centrifugation at 2500 rpm for 10 min followed by filtration with 0.45 μm filter and the virus concentrated by ultracentrifugation on a sucrose cushion as described before (Wickersham et al., 2007a).

Rabies viruses pseudotyped with oG were produced infecting BHK-TGoG cells in 10 cm dishes with 1 mL of viral supernatant. Cells were split the day after infection and maintained for one or two days at 37°C and 5% CO_2_ checking daily the viral spreading. When 70%–80% of cells expressed the viral marker, the media was replaced with 2% FBS DMEM and maintained for 2-3 days at 35°C and 3% CO_2_. Then, the supernatant was collected and processed as previously described.

Rabies viruses pseudotyped with EnvA were produced as previously described (Wickersham et al., 2007a) using BHK-T-EnvA cells instead of BHK-EnvA cells.

#### In vitro cytotoxicity analysis

Human Embryonic Stem cells (hESCs) derived neurons were kindly provided by Dr. Rick Livesey. Cells were plated in 24-wells glass bottom plates and infected over-night with attenuated or control ΔG-Rabies viruses supernatants at comparable MOI to obtain ∼5% of infected cells. Cells were imaged every 4 days post infection overnight in a 37°C heated Leica SP8 confocal microscope in Hibernate®-A Medium (Invitrogen) with 5 random fields imaged for each well. Cells survival was calculated normalizing each condition to the mortality of control Lentivirus^GFP^ infected hESCs derived neurons imaged and processed in the same conditions.

#### Immunohistochemistry

Mice were perfused with ice cold phosphate buffered saline (PBS) followed by 4% paraformaldehyde (PFA) in PBS. Brains were incubated in PFA overnight at 4°C, rinsed twice with PBS and then dehydrated for 48 hr in 30% sucrose in PBS at 4°C. The brains were frozen in O.C.T. compound (VWR) and sliced at the cryostat (Leica, Germany). Free-floating sections were rinsed in PBS and then incubated in blocking solution (1% bovine serum albumin and 0.3% Triton X-100 in PBS) containing primary antibodies for 24 hr at 4°C. Sections were washed with PBS three times and incubated for 24 hr at 4°C in blocking solution with secondary antibodies. Immuno-labeled sections were washed three times with PBS and mounted on glass slides. Antibodies used in this study were chicken anti-GFP (Thermo Scientific, A10262, 1:1000), rabbit anti-RFP (Rockland, 600-401-379, 1:2000), donkey anti-chicken alexa 488 (Jackson ImmunoResearch, 703-545-155, 1:1000), donkey anti-rabbit Cy3 (Jackson ImmunoResearch, 711-165-152, 1:1000) and rabbit anti-cleaved caspase-3 (NEB, #9661, 1:500).

#### Viral injections

All procedures using live animals were approved by the Home Office and the LMB Biosafety committee. For all experiments mice aged between 6-12 weeks were used. Mice were anesthetized with isofluorane delivered at a flow of 3% in 2L/min of oxygen for the initial induction and then maintained at 1%–2% in 2 L/min of oxygen. The anesthetized animal was placed into a stereotaxic apparatus (David Kopf Instruments) and Rimadyl (2 mg/kg body weight) was administered subcutaneously (s.c.) as anti-inflammatory. A small hole (500 μm diameter) was drilled and viruses were injected using a Hamilton neurosyringe. The syringe was left in the brain for 5 min before being retracted. Viruses were injected at the following titers: 3x10^8^ infectious units/ml for Rabies viruses, 2x10^12^ genomic copies/ml for AAVs, 3x10^8^ infectious units/ml for Lentiviruses. Up to a max of 400 nL in volume of virus were injected in the following brain areas: CA1 (AP: −2.3 mm, ML: 1.65 mm and DV: 1.45mm from bregma), V1 (AP: −3.3 mm, ML: 2.8 mm and DV: 0.25 mm from brain surface), V2 (AP: −3.6 mm, ML: 1.2 mm, DV:0.6 mm from bregma), NAc (AP: 1.3 mm, ML: 1.3 mm and DV: 4.75 mm from bregma), VTA (AP: −3.2 mm, ML: 0.45 mm and DV: 4.35 mm from bregma), BLA (AP: −1.65 mm, ML: 2.9 mm and DV: 4.7 mm from bregma).

#### In vivo cytotoxicity analysis

To test the in vivo viral cytotoxicity, 400 nL of same titer (3x10^8^ infectious units/ml) attenuated and control ΔG-Rabies were injected in CA1 area of the hippocampus in both hemispheres. At 1-2-3 weeks or 2-6 months p.i. brains were collected and sectioned at the cryostat (35 μm). Infected neurons were imaged sampling the entire hippocampus (acquiring one every 4 sections) using a robot assisted Nikon HCA microscope mounting a 10x (0.45NA) air objective and fluorescent hippocampal neurons counted using Nikon HCA analysis software. Cell survival for attenuated and control ΔG-Rabies was calculated normalizing each time point to the mortality of control Lentivirus-GFP infected hippocampi using the same injection protocol.

To confirm no cytotoxic effect upon SiR infection in hippocampus, brains slices at 1 and 2 weeks p.i. were stained with anti-cleaved caspase-3 (cCaspase3, 1:500). Confocal images were acquired using a Leica SP8 confocal microscope mounting a 20X (0.75NA) air objective and the total number of cCaspase3 positive neurons counted in PBS, SiR or ΔG-Rabies injected hippocampi.

#### Drug induced reactivation of SiR virus in vivo

*Rosa-LoxP-STOP-LoxP-YFP* animals were injected in CA1 of hippocampus with an AAV expressing constitutively rtTA and TEV protease under the control of a doxycycline inducible promoter (Table S1). 1-week p.i. the same area was re-injected with SiR^CRE-mCherry^ and doxycycline (Santa Cruz Biotechnology, 100 mg/Kg) administered by oral gavage at 1 or 2 weeks post SiR injection. 1 week after drug administration brains were collected and sectioned at the cryostat (35 μm). Infected neurons were imaged and counted sampling the entire hippocampus (acquiring one every 4 sections) using a robot assisted Nikon HCA microscope.

#### Transsynaptic and intraneuronal retrograde tracing

To test the in vivo retrograde transsynaptic viral spreading efficiency, 400 nL of AAV^TVA-oG^ were injected in the NAc contralateral in the 2 hemispheres of *Rosa-LoxP-STOP-LoxP-tdTomato* animals. At 3 weeks post-AAV injection, TVA expressing cells were targeted in the 2 hemispheres with 400 nL of same titer (3x10^8^ infectious units/ml) SiR^CRE^ or control ΔG-Rabies^GFP^. At 1 and 3 weeks p.i. brains were collected and sectioned at the cryostat (35 μm). Transsynaptic spreading and neuronal survival were assessed by imaging SiR or ΔG-Rabies infected neurons in the NAc, BLA and VTA (acquiring one every 2 sections) using a robot assisted Nikon HCA microscope mounting a 10x (0.45NA) air objective and ipsilateral fluorescent neurons counted using Nikon HCA analysis software. Transsynaptic spreading efficiency was calculated by dividing the number of inputs neurons in the BLA or VTA by the number of starting neurons and normalized to SiR efficiency.

To test the in vivo intraneuronal retrograde viral efficiency, 400 nL of SiR^CRE^ (8.5x10^8^ infectious units/ml) or 400 nL of rAAV2-retro^nucGFP^ (4x10^12^ genomic copies/ml) were injected in the VTA of *Rosa-LoxP-STOP-LoxP-tdTomato* mice.

At 1 and 3 weeks p.i., for SiR and rAAV2-retro respectively, brains were collected and sectioned at the cryostat (35 μm). Retrograde spreading was assessed by imaging SiR and ΔG-Rabies infected neurons in the NAc, mPFC and LH (acquiring one every 4 sections) using a robot assisted Nikon HCA microscope mounting a 10x (0.45NA) air objective and fluorescent neurons were counted using Nikon HCA analysis software.

#### Analysis of SiR genomic copies in vivo

To evaluate the genomic copies of SiR virus in the infected animals over time, SiR^CRE-mCherry^ was injected in the CA1 region of hippocampus of *Rosa-LoxP-STOP-LoxP-YFP* animals. After 1, 2, 3 or 8 weeks, mice were culled and the injected hippocampi manually dissected immediately after. The hippocampi were homogenized using a Tissuelyser II (QIAGEN) and processed accordingly to manufactory instruction with RNeasy kit (QIAGEN) to extract total RNA. 500 ng of RNA per hippocampus were retrotranscribed using superscript IV kit (Invitrogen) and analyzed for *GADPH*, *YFP* and *mCherry* expression by quantitative PCR (rotorgene sybr-green). The Livak method was applied for quantification: the expression of *YFP* and *mCherry* was normalized to the expression of the *GADPH* housekeeping gene (ΔCT = CT_gene_ - CT_GADPH_) and the variation over time as fold change (2^-ΔΔCT^) to the 1 week time point (ΔΔCT = ΔCT_Time point_ – ΔCT_1 week_).

#### Electrophysiology

For electrophysiological recordings, SiR^CRE-mCherry^ was injected bilaterally in the CA1 of one month-old *Rosa-LoxP-STOP-LoxP-tdTomato* or *Rosa-LoxP-STOP-LoxP-ChR2YFP* mice. Recordings were made either one week or 2 and 5 months p.i.

Coronal hippocampal slices (350 μm) were prepared using a vibrating microtome (7000smz-2, Campden Instruments LTD, Loughborough, UK) in ice-cold sucrose-based cutting solution oxygenated with carbogen gas (95% O_2_, 5% CO_2_) and with the following composition (in mM): KCl 3, NaH_2_PO_4_ 1.25, MgSO_4_ 2, MgCl_2_ 1, CaCl_2_ 1, NaHCO_3_ 26.4, glucose 10, sucrose 206, ascorbic acid 0.40, kynurenic acid 1. Slices were incubated at 37°*C* for 30 min in a submerged-style holding chamber with oxygenated artificial cerebrospinal fluid (aCSF; in mM: NaCl 126, KCl 3, NaH_2_PO_4_ 1.25, MgSO_4_ 2, CaCl_2_ 2, NaHCO_3_ 26.4, glucose 10) with an osmolarity adjusted to 280-300 mOsm/L and stored thereafter in the same holding chamber at room temperature for at least 1 hr. Slices were then individually transferred to the recording chamber and were perfused with oxygenated aCSF at room temperature at a flow-rate of approximately 2 mL/min.

Whole-cell current-clamp recordings were obtained from CA1 neurons using 6-9 MΩ pipettes pulled from borosilicate glass capillaries (1.5 mm OD x 0.86 mm ID). Pipettes were filled with artificial intracellular solution containing (in mM): K-gluconate 150, HEPES 10, NaCl 4, ATP-Mg 4, GTP-Na 0.3 and EGTA 0.2; adjusted to pH 7.2 and osmolarity 270-290 mOsm/L. Data were recorded using an Axon Multiclamp 700B amplifier (Molecular Devices, Union City, CA, USA) and signals were low-pass filtered at 2 kH and acquired at 5 kHz using a digitizer (Axon Digidata 1550A, Molecular Devices, Union City, CA, USA) on a PC running pClamp. Light-evoked responses from neurons infected with SiR virus were elicited using a 450-490 nm LED light (pE-300 coolLED system, Scientifica Ltd, Uckfield, UK) through a 40X water immersion objective (0.8 NA).

In Figure 5K, putative postsynaptic neurons were pre-screened on the basis of being mCherry^OFF^ and in proximity of presynaptic mCherry^ON^. Latencies from the onset of the optical stimulation were then assessed.

#### Pharmacology

The AMPA receptor antagonist DNQX (20 μM) was used in a subset of electrophysiological recordings in order to probe synaptic connectivity between neurons infected with SiR virus and neighboring neurons.

#### In vivo 2-photon imaging

Injected *Rosa-LoxP-STOP-LoxP-tdTomato* mice (see *Viral injections* section) were anesthetized with isofluorane 2%. Animal pinch withdrawal and eyelid reflex were tested to assay the depth of anesthesia. Rimadyl (2 mg/kg body weight) was injected subcutaneously as an anti-inflammatory. Both eyes were covered with an eye ointment to prevent corneal desiccation during the experiment. The animal was head-fixed and a metal head-post cemented to the skull. A craniotomy of 4 mm in diameter was drilled over the V1 cortex. After the removal of the skull, the cortical surface was kept moist with a cortex buffer, containing: 125 mM NaCl, 5 mM KCl, 10 mM Glucose, 10 mM HEPES, 2 mM MgSO_4_ and 2 mM CaCl_2_, adjusted to pH 7.3. The cortex was then covered with a custom made plug coverslip (Goldey et al., 2014) and sealed with Super Glue and dental cement. Mice were anesthetized with 2% of isofluorane and mounted under a two-photon laser-scanning microscope (Multiphoton Imaging System, Scientifica, Uckfield, United Kingdom) equipped with a Ti:sapphire mode-locked laser (Mai Tai-Series, Spectra Physics) tuned at 920 nm. Imaging was performed through a water-immersion lens (Nikon, 16X, 0.8 NA) at a resolution of 256 × 256 pixels with zoom 2 or 4, leading to a field of view of 390 × 390 μm and 195 × 195 μm respectively. Data were acquired at 3.5 Hz. The objective was shielded with a black fabric cone equipped with a plastic o-ring fixed onto the head plate (Andermann et al., 2010). Visual stimulation was controlled using a custom-made GUI in Python (based on PsychoPy toolbox) and was performed with a LED screen positioned 15 cm from the left eye of the mouse. Moving square-wave gratings were presented at 12 directions in 30 degrees steps and a photodiode was used to detect the starting and the ending time of each stimulus. Each grating direction was presented 5 times in random order alternated with a blank condition. The spatial frequency of the grating was 0.04 cycles per degree (cpd) and the temporal frequency was 1 Hz. Imaging and visual stimulation were triggered together using Arduino micro-controller board. Imaging session lasted up to 2 hr and the power at sample was controlled in the range 30-40 mW. Data analysis was performed in ImageJ and MATLAB and was restricted to cell bodies. Detection of region of interest (ROI) was performed with Suite2p (Pachitariu et al., 2016). The relative changes in fluorescence were calculated as dF/F_0_ = (F(t)-F_0_)/(F_0_). Orientation tuning curves were generated by taking the mean response for each orientation during the entire stimulus period. Response amplitudes are presented as the relative change in fluorescence during the stimulus period compared to the pre-stimulus baseline (dF/F). All data are presented as mean ± SEM.

### Quantification and Statistical Analysis

Mean values are accompanied by SEM. No statistical methods were used to predetermine sample sizes. Data collection and analysis were not performed blind to the conditions of the experiments. Statistical analysis was performed in R and/or MATLAB. Paired t test and one-way ANOVA test were used to test for statistical significance when appropriate. Statistical parameters including the exact value of n, precision measures (mean ± SEM) and statistical significance are reported in the text and in the figure legends (see individual sections). The significance threshold was placed at α = 0.05.

## Author Contributions

M.T. and E.C. conceived the project and designed the experiments. E.C. performed all the experiments with the exception of the electrophysiological recordings. F.M. provided technical support for viral production, cell culture, and molecular biology. L.M. performed the in vivo calcium imaging with the help of E.C. A.G.-R. performed the electrophysiological recordings. E.C., A.G.-R., L.M., and M.T. analyzed the data. E.C., M.T., A.G.-R., and L.M. prepared the figures. M.T. wrote the manuscript with E.C. incorporating feedback from L.M., F.M., and A.G.-R.

## Supplemental Information

Document S1. Tables S1 and S2

Movie S1. Two-Photon Ca^2+^ Imaging after 1 Month from SiR Injection, Related to Figure 6 Movie illustrates the Ca2+ signal dynamics while presenting 12 different moving gratings (same field of view of Figure 4B). Frame rate: 40/s. Acquisition time frame: 3.5 Hz. Scale bar, 50 μm.
