# Supplementary material for: Life-Long Genetic and Functional Access to Neural Circuits Using Self-Inactivating Rabies Virus
Source: Cell. 2017 Jul 13;170(2):382–392.e14. doi: 10.1016/j.cell.2017.06.014 (PMC5509544; doi:10.1016/j.cell.2017.06.014)
Supplement: Document S1. Tables S1 and S2 [file mmc1.pdf]

Cell, Volume *170*

Supplemental Information

Life-Long Genetic and Functional Access
to Neural Circuits Using Self-Inactivating
Rabies Virus

Ernesto Ciabatti, Ana González-Rueda, Letizia Mariotti, Fabio Morgese, and Marco Tripodi

**Rabies**	$_{\text{N-ter}}$**TAGs**	$_{\text{C-ter}}$**PEST**
pSAD-F3-mCherry	-	-
pSAD-F3-$_{\text{N-ter}}$TAGs-mCherry	N^{Myc}–P^{FLAG}–M^{V5}	-
pSAD-F3-$_{\text{N-ter}}$TAGs-N^{PEST}-mCherry	N^{Myc}–P^{FLAG}–M^{V5}	N^{PEST}
pSAD-F3-$_{\text{N-ter}}$TAGs-M^{PEST}-mCherry	N^{Myc}–P^{FLAG}–M^{V5}	M^{PEST}
pSAD-F3-$_{\text{N-ter}}$TAGs-P^{PEST}-mCherry	N^{Myc}–P^{FLAG}–M^{V5}	P^{PEST}
pSAD-F3-$_{\text{N-ter}}$TAGs-L^{PEST}-mCherry	N^{Myc}–P^{FLAG}–M^{V5}	L^{PEST}
pSAD-F3-N^{PEST}-mCherry (**SiR**$^{\textbf{mCherry}}$)	-	N^{PEST}
pSAD-F3-$_{\text{N-ter}}$TAGs-$(P+L)^{PEST}$-mCherry	N^{Myc}–P^{FLAG}–M^{V5}	P^{PEST} L^{PEST}
pSAD-F3-$(P+L+N)^{PEST}$-mCherry	-	P^{PEST} L^{PEST} N^{PEST}
pSAD-F3-N^{PEST}-iCRE-2A-mCherryPEST (**SiR**$^{\textbf{CRE-mCherry}}$)	-	N^{PEST}
pSAD-F3-N^{PEST}-$_{\text{C}}$TEVp-FKBP-2A-FRB-$_{\text{N}}$TEVp-iCRE	-	N^{PEST}
pSAD-F3-N^{PEST}-FLPo (**SiR**$^{\textbf{FLP}}$)	-	N^{PEST}
**Lentiviruses**	**Gene A**	**Gene B**
pLenti-$_{\text{H2B}}$GFP-2A-GlySAD	$_{\text{H2B}}$GFP	GlySAD
pLenti-puro-2A-TEVp	Puromycin N-Acetyl-Transferase (PAC)	TEVp
pLenti-GFP	GFP	-
pLenti-$_{\text{H2B}}$GFP-2A-oG	$_{\text{H2B}}$GFP	oG
**AAVs**	**Gene A**	**Gene B**
AAV-CMV-TVAmCherry-2A-G	TVAmCherry	G
AAV-TRE_{tight}-TEVp-CMV-rtTA	TEVp (doxy dependent)	rtTA
AAV-CAG-GCaMP6s	GCaMP6s	-
AAV-CAG-FRT-$_{\text{H2B}}$GFP	$_{\text{H2B}}$GFP (FLP dependent)	-
AAV-CMV-FLEX-TVAmCherry-2A-oG	TVAmCherry (CRE dependent)	oG (CRE dependent)

Table S1.

List of viral constructs generated for the study, Related to Figure 1

	Rosa-LoxP-STOP-LoxP-tdTomato **mice**				*Rosa-LoxP-STOP-LoxP-ChR2YFP* **mice**			
	1 week p.i.		5 months p.i.		1 week p.i.		2 months p.i.	
	SiR+ tdTom+ n = 14	CTR n =14	SiR+ tdTom+ n = 10	CTR n = 9	SiR + ChR2 + n = 17	CTR n = 15	SiR + ChR2 + n = 19	CTR n = 17
**RMP (mV)**	-60.4 ± 0.9	-58.4 ± 1.0	-61.8 ± 1.5	-62.8 ± 1.1	-57.8 ± 0.6	-59.1 ± 1.1	-57.0 ± 1.0	-57.2 ± 0.9
**Input Resistance (MΩ)**	274 ± 28	267 ± 24	250 ± 26	260 ± 43	249 ± 15	249 ± 23	274 ± 24	290 ± 24
**AP amplitude (mV)**	102.9 ± 3.0	99.4 ± 3.2	99.8 ± 3.3	99.7 ± 3.0	98.6 ± 1.9	101.5 ± 1.7	97.8 ± 2.6	99.7 ± 3.0
**AP half-width (ms)**	2.0 ± 0.1	2.0 ± 0.1	2.0 ± 0.1	2.0 ± 0.1	2.3 ± 0.1	2.1 ± 0.1	2.3 ± 0.1	2.2 ± 0.1
**AP threshold (mV)**	-45.3 ± 1.6	-45.7 ± 0.9	-45.9 ± 1.1	-46.1 ± 1.0	-45.3 ± 0.8	-45.8 ± 1.1	-43.9 ± 1.4	-43.6 ± 1.7

Table S2

Electrophysiological properties of SiR infected neurons compared to neighboring non-infected neurons recorded in Rosa-*LoxP-STOP-LoxP-tdTomato* and Rosa-*LoxP-STOP-LoxP-ChR2YFP* mice, Related to Figure 5
