# Supplementary figures and images for: Life-Long Genetic and Functional Access to Neural Circuits Using Self-Inactivating Rabies Virus
Source: Cell. 2017 Jul 13;170(2):382–392.e14. doi: 10.1016/j.cell.2017.06.014 (PMC5509544; doi:10.1016/j.cell.2017.06.014)

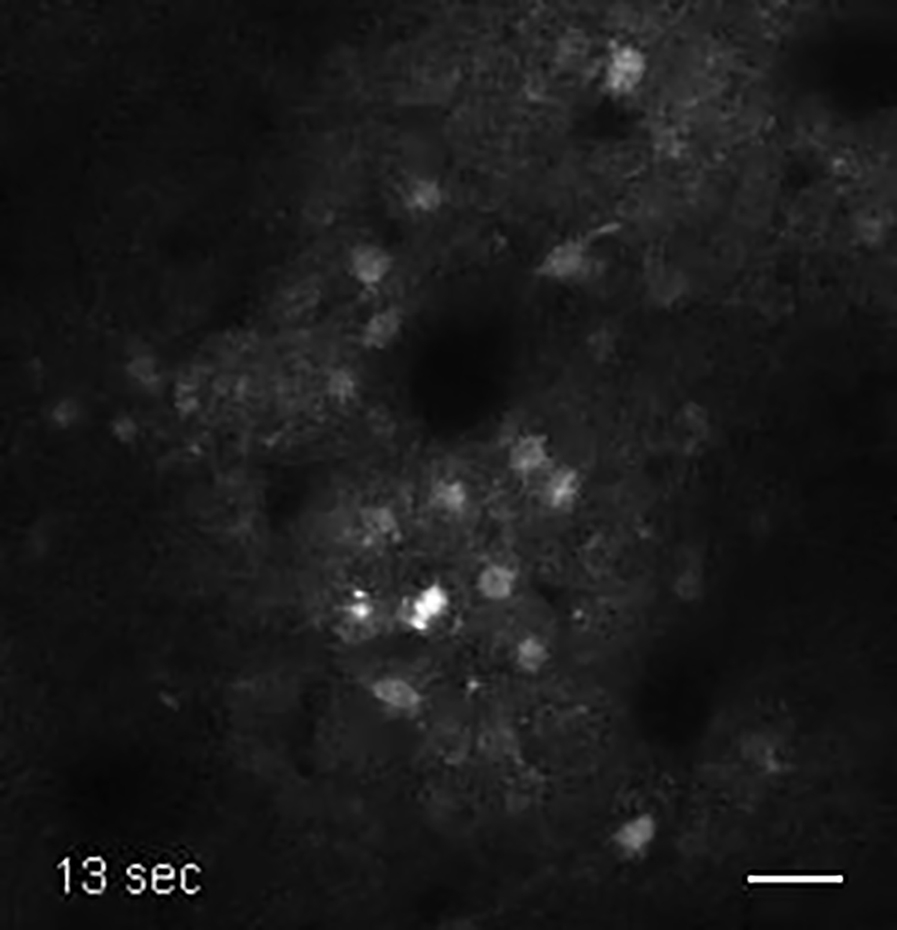

Supplement: Movie S1. Two-Photon Ca2+ Imaging after 1 Month from SiR Injection, Related to Figure 6 — Movie illustrates the Ca2+ signal dynamics while presenting 12 different moving gratings (same field of view of Figure 4B). Frame rate: 40/s. Acquisition time frame: 3.5 Hz. Scale bar, 50 μm. [file mmc2.jpg]
